# Ceria‐Supported Cobalt Catalyst for Low‐Temperature Methanation at Low Partial Pressures of CO_2_

**DOI:** 10.1002/anie.202214864

**Published:** 2022-12-22

**Authors:** Job J. C. Struijs, Valery Muravev, Marcel A. Verheijen, Emiel J. M. Hensen, Nikolay Kosinov

**Affiliations:** ^1^ Laboratory of Inorganic Materials and Catalysis Department of Chemical Engineering and Chemistry Eindhoven University of Technology P.O. Box 513 5600MB Eindhoven The Netherlands; ^2^ Department of Applied Physics Eindhoven University of Technology P.O. Box 513 5600MB Eindhoven The Netherlands; ^3^ Eurofins Material Science Netherlands BV 5656AE Eindhoven The Netherlands

**Keywords:** Atmospheric CO_2_ Valorization, CO_2_ Hydrogenation, Ceria, Cobalt, Reaction Mechanisms

## Abstract

The direct catalytic conversion of atmospheric CO_2_ to valuable chemicals is a promising solution to avert negative consequences of rising CO_2_ concentration. However, heterogeneous catalysts efficient at low partial pressures of CO_2_ still need to be developed. Here, we explore Co/CeO_2_ as a catalyst for the methanation of diluted CO_2_ streams. This material displays an excellent performance at reaction temperatures as low as 175 °C and CO_2_ partial pressures as low as 0.4 mbar (the atmospheric CO_2_ concentration). To gain mechanistic understanding of this unusual activity, we employed in situ X‐ray photoelectron spectroscopy and operando infrared spectroscopy. The higher surface concentration and reactivity of formates and carbonyls—key reaction intermediates—explain the superior activity of Co/CeO_2_ as compared to a conventional Co/SiO_2_ catalyst. This work emphasizes the catalytic role of the cobalt‐ceria interface and will aid in developing more efficient CO_2_ hydrogenation catalysts.

## Graphical abstract

Co/CeO_2_ is an efficient low‐temperature methanation catalyst at CO_2_ partial pressures down to atmospheric concentrations. Advanced spectroscopic investigations unveiled that the combination of a higher concentration and higher intrinsic activity of active intermediates (formates and carbonyls) explains the high activity, emphasizing the role of the metal‐ceria interface.

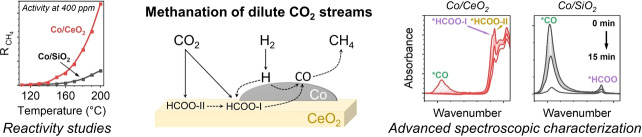

## Introduction

The atmospheric carbon dioxide concentration is increasing at an accelerating pace. Mitigation of the negative consequences from CO_2_ emissions, i.e. climate change, is a significant challenge.^[1]^ Carbon dioxide capture followed by its utilization via catalytic hydrogenationto useful chemical building blocks and fuels is a promising solution to this problem.[^1^, ^2^] Methane, being the most favored hydrogenation product thermodynamically, is a suitable molecule for CO_2_ utilization.^[3]^ High yields of methane can be achieved even under atmospheric pressure, in contrast to other hydrogenation products—methanol and higher hydrocarbons/oxygenates.[^4^, ^5^] Moreover, methane is compatible with the existing natural gas transport and distribution infrastructure.^[6]^ Importantly, the methanation of CO_2_ (Sabatier reaction) is a promising process for the storage of renewable energy^[7]^ and the removal of CO_*x*_ from hydrogen‐rich streams (needed for ammonia production and fuel cells).^[8]^ Lastly, methanation can be used to upgrade biogas streams to a high‐quality synthetic natural gas by converting CO_*x*_ and increasing the methane content.^[6]^ The efficiency and practical applicability of all these processes depend on the activity, selectivity, and stability of methanation catalysts.

Many transition metals can catalyze CO_2_ methanation. High activity and stability in a broad temperature range is achieved over costly noble metals (e.g., Ru and Rh).^[4]^ More affordable and extensively studied Ni catalysts demonstrate a limited activity in low‐temperature (<200 °C) CO_2_ methanation (Figure S1). Cobalt is an alternative base metal catalyst for the low‐temperature methanation, although Co catalysts with high activity below 200 °C are yet to be identified (Figure S1). Furthermore, to date, numerous studies focused on the effect of increasing the reactant partial pressures on the performance of CO_2_ catalysts.^[9]^ On the contrary, the reaction at a low partial pressure of CO_2_ remains largely underexplored, especially for heterogeneous systems (Figure S2).^[10]^ This aspect is increasingly important for the direct valorization of atmospheric CO_2_, with no separate energy‐intensive separation and purification steps, which can offer environmental and economic benefits.[^11^, ^12^] Kuramoto and co‐workers, for example, demonstrated the feasibility of such a process in a cycled fixed‐bed reactor using a hybrid Ni/Na‐γ‐Al_2_O_3_ system, fulfilling both the roles of CO_2_ adsorbent and methanation catalyst.^[13]^

Generally the activity and selectivity of methanation catalysts strongly depend on the support material and the nature of the metal‐support interactions (MSI).[^8^, ^14^] For example, redox properties,^[15]^ charge transfer effects,^[16]^ and formation of metal‐support interfacial sites[^3^, ^17^] were reported to influence the catalytic performance of methanation catalysts. Catalysts supported by the redox‐active cerium dioxide (CeO_2_, ceria) are particularly active in CO_2_ methanation.[^18^, ^19^] The abundant basic sites in addition to the high reducibility of ceria, easily forming oxygen vacancies, were reported to result in a strong adsorption and subsequent activation of CO_2_.^[20]^ Recently we demonstrated how the performance of ceria‐based catalysts can be optimized through engineering of the Co‐CeO_2_ interface.^[8]^

In this work, we demonstrate that Co/CeO_2_ can efficiently catalyze low‐temperature CO_2_ methanation at low partial pressures of CO_2_. Co/CeO_2_ displays a high CO_2_ methanation activity at CO_2_ partial pressures as low as 0.4 mbar (corresponding to the atmosphere concentration of 400 ppm). Using transient kinetic step‐response and steady‐state isotopic transient kinetic analysis (SSITKA), followed by Fourier‐transform infrared (FTIR) spectroscopy, we identified formate and carbonyl species as the key reaction intermediates. Operando FTIR spectroscopy demonstrated the presence of two types of formate species on Co/CeO_2_. Both formates contribute to the formation of carbonyls but with a different rate. Furthermore, an additional linear carbonyl species, assigned to the adsorption of CO next to adsorbed carbon atoms, was identified. Lastly, the formation of oxygen vacancies and cerium hydrides during the reduction process of Co/CeO_2_ was observed by near‐ambient pressure X‐ray photoelectron spectroscopy (NAP‐XPS). The combination of higher reactivity of formates and carbonyls and the higher concentration of formates on Co/CeO_2_, as compared to a reference Co/SiO_2_ catalyst, explains the high activity of the ceria‐based catalyst at a low partial pressure of CO_2_ and low temperature. The mechanistic findings of this work emphasize and clarify the role of the cobalt‐ceria interface and will help developing better catalysts for CO_2_ hydrogenation.

## Results and Discussion

### CO_2_ Hydrogenation Activity of Co/CeO_2_

**Figure 1:**
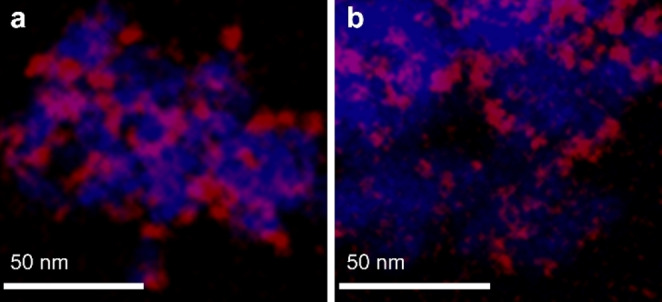
TEM‐EDX mapping of a) Co/CeO_2_ and b) Co/SiO_2_. Cobalt particles are shown in red; the support in blue.

A Co/CeO_2_ and a Co/SiO_2_ catalyst (9 wt %, Table S2) were prepared by strong electrostatic adsorption of an in situ formed hexaamminecobalt(III) complex ([Co(NH_3_)_6_]^3+^) in wet impregnation mode. After calcination and reduction, the metal particle sizes for both catalysts were close to 7 nm, according to TEM and CO chemisorption (Table S2 and Figure S3). The even distribution of Co over both supports was confirmed by TEM‐EDX (Figure 1).

**Figure 2:**
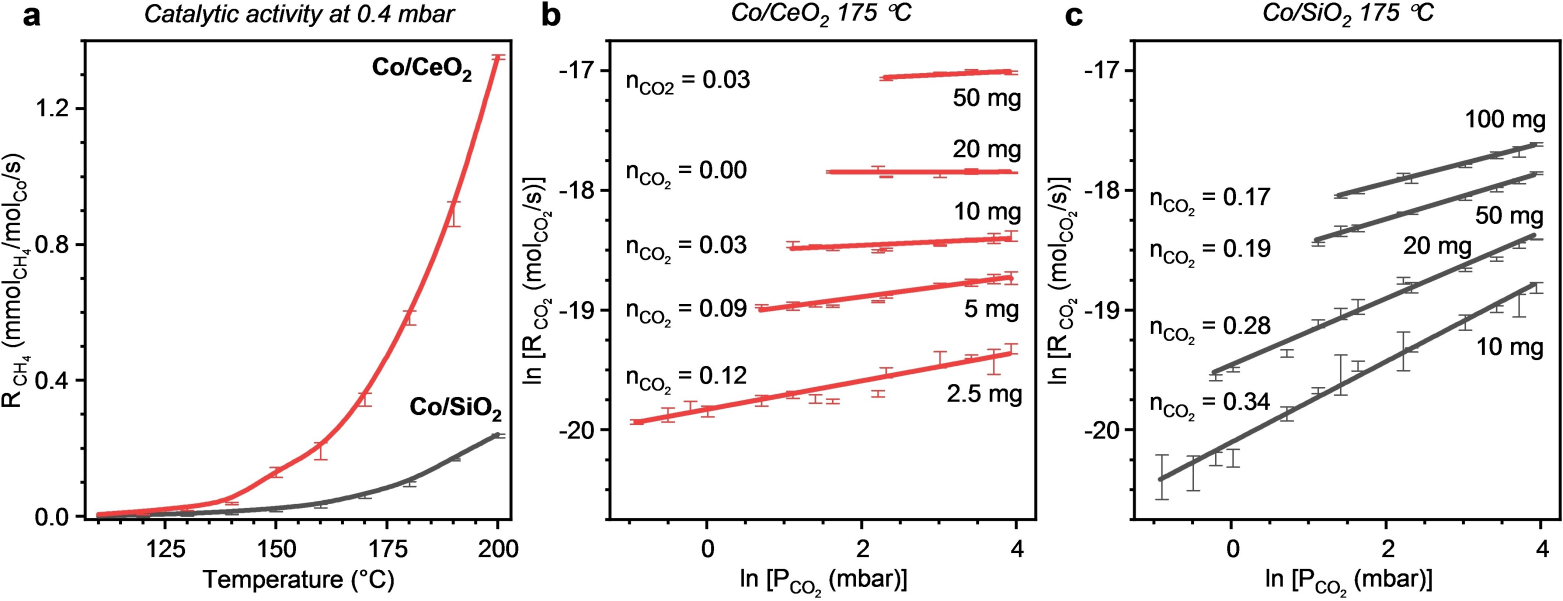
The catalytic activity of Co/CeO_2_ and Co/SiO_2_ in CO_2_ hydrogenation. a) Rate of CH_4_ formation as a function of temperature at 0.4 mbar of CO_2_. Error bars indicate the standard deviation of measurements with different catalyst loadings (CO_2_ conversions <30 %). b), c) Reaction orders with respect to CO_2_ (*n* ${}_{\mathrm{CO}{}_{2}}$ ±0.01) for Co/CeO_2_ (b) and Co/SiO_2_ (c) at 175 °C with CO_2_ partial pressures of 0.4–5 mbar. The data of b&c expressed as specific reaction rates can be found in Figure S4. Error bars indicate the standard deviation between GC measurements (CO_2_ conversions <10 %, except 2.5 mg Co/CeO_2_ at 400 ppm). Conditions: 2.5–100 mg catalyst, 0.4–5 mbar CO_2_ and 200 mbar H_2_ in He, total flow 200 mL min^−1^.

After in situ reduction in hydrogen at 500 °C, the catalytic performance of the cobalt nanoparticles deposited on a reducible (CeO_2_) and non‐reducible (SiO_2_) support was assessed (Figure 2). The catalytic activity was tested as a function of temperature at a low CO_2_ partial pressure of 0.4 mbar (Figure 2a). Co/CeO_2_ demonstrates a significantly higher activity than Co/SiO_2_ at each temperature. Additionally, the prepared Co/CeO_2_ catalyst demonstrates a higher low‐temperature (<200 °C) catalytic activity than previously reported Co and Ni catalysts (Figure S1).

In order to understand the difference in CO_2_ activation between the studied catalysts, the reaction orders with respect to CO_2_ were determined at 175 °C (Figure 2b,c). The partial pressure of CO_2_ was varied by two orders of magnitude—from 0.4 to 50 mbar. Over the whole pressure range, the Co/CeO_2_ catalyst displays a much higher metal‐normalized activity compared to Co/SiO_2_ and previously reported Co and Ni catalysts (Figure S2). The methanation at low partial pressures of CO_2_ over Co/CeO_2_ proceeds with >95 % selectivity to CH_4_ (Figure S5). Under the same reaction conditions, the CH_4_ selectivity over Co/SiO_2_ was much lower. Interestingly, the reaction order in CO_2_ is higher for Co/SiO_2_ (Figure 2b,c). The near zero reaction orders for Co/CeO_2_ demonstrate that the coverage of CO_2_ during steady‐state reaction is sufficiently high in the whole pressure range. This finding is in line with the ability of the CeO_2_ support in efficiently capturing CO_2_ from dilute streams.^[20]^

### Operando FTIR Spectroscopy at Low and High Partial Pressures of CO_2_

The activity of heterogeneous catalysts is governed by the transformation of surface intermediates.^[14]^ To investigate the surface species present on the Co/CeO_2_ and Co/SiO_2_ catalysts during CO_2_ methanation, operando FTIR spectroscopy was applied. Similar to the activity measurements, the experiments were performed at 175 °C under steady‐state conditions, as confirmed by mass spectrometry and FTIR (e.g., Figures S6, S7). The catalysts were reduced in H_2_ at 500 °C and subsequently exposed to a continuous flow of CO_2_+H_2_ (200 mL min^−1^) with either a high (25 mbar) or low (0.6 mbar) CO_2_ partial pressure at 175 °C.

**Figure 3:**
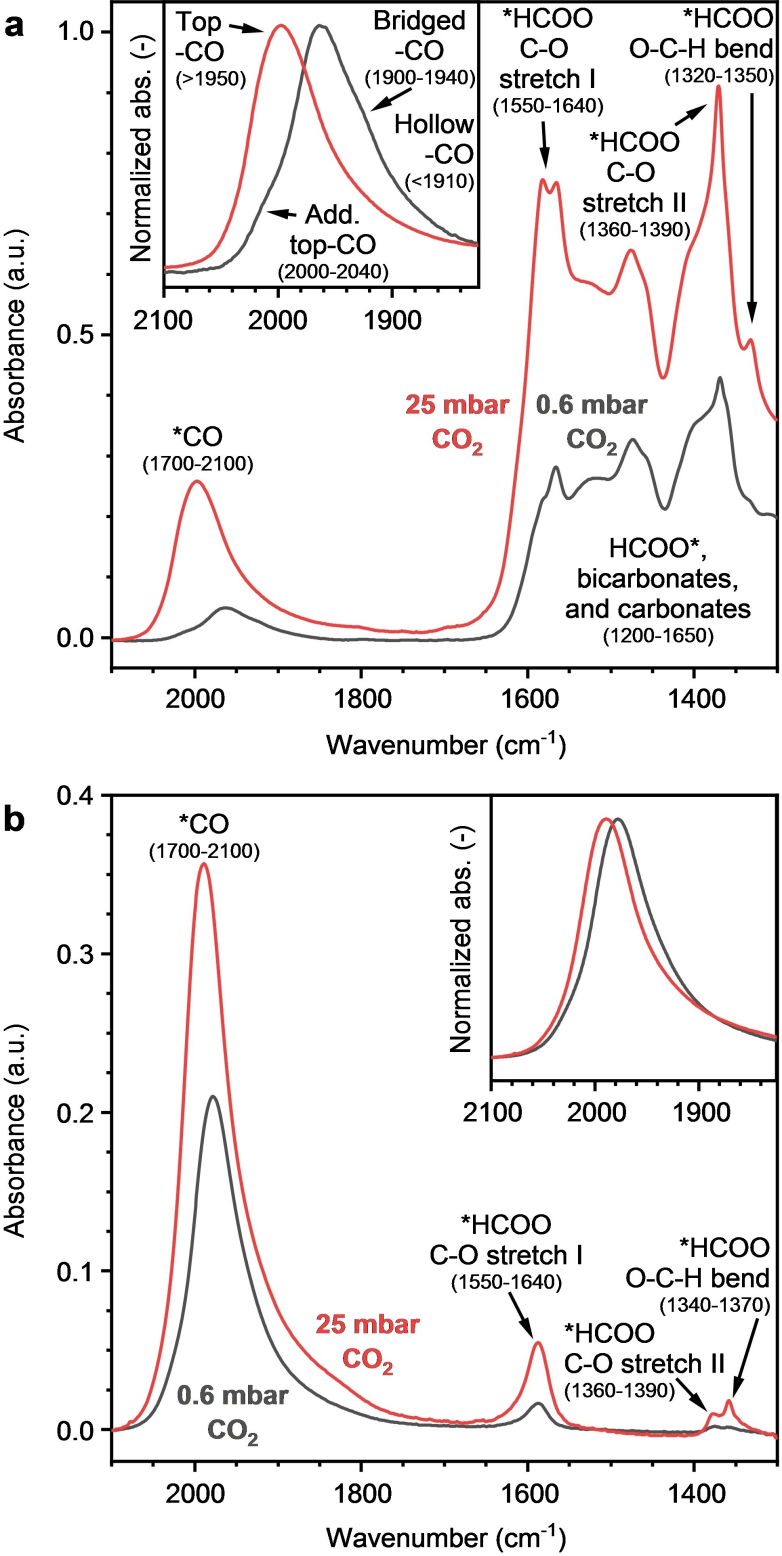
Steady‐state FTIR spectra at 0.6 mbar and 25 mbar CO_2_ for a) Co/CeO_2_ and b) Co/SiO_2_. Several vibrations are indicated, including their approximate positions in cm^−1^. The insets show the intensity‐normalized carbonyl signals. All spectra were normalized by pellet weight. Conditions: 175 °C, 0.6 or 25 mbar CO_2_/200 mbar H_2_ in He, total flow 200 mL min^−1^.

At both partial pressures (Figure 3), the characteristic bands located between 1700–2100 cm^−1^ can be assigned to carbonyls adsorbed on metallic cobalt (*CO).^[21]^ Bands centered at ≈1950 cm^−1^ and higher are assigned to linear adsorption of CO (top‐CO).^[22]^ Bands located below ≈1950 cm^−1^ are assigned to the bridged and hollow adsorption modes of CO (bridged‐CO and hollow‐CO).[^21^, ^23^] Two different C−O stretch vibrations assigned to formate species were observed between 1550–1640 cm^−1^ (C−O stretch I) and 1360–1390 cm^−1^ (C−O stretch II). The C−H stretch (2800–2890 cm^−1^) and O−C‐H bending (≈1320–1370 cm^−1^) formate vibrations were also identified.

Bicarbonates and multiple carbonate species were present on Co/CeO_2_ as manifested by several bands in the regions of 1200–1650 cm^−1^ and 950–1100 cm^−1^ (Figure 3a).[^3^, ^24^] The much higher surface coverages of formates, bicarbonates, and carbonates compared to Co/SiO_2_ confirm the ability of ceria to strongly adsorb CO_2_.^[4]^ Previously, it was proposed that these species are the precursors that lead to the formation of carbonyl species and eventually methane formation on alumina‐ and ceria‐supported catalysts.[^3^, ^25^]

The observed difference in the concentration of surface species at 0.6 and 25 mbar (Figure 3, discussed in Note S1) indicates the importance of studying the catalysts under the relevant reaction conditions, in this case low CO_2_ partial pressure.^[26]^ At 0.6 mbar of CO_2_, an additional top‐CO peak is observed as a blue‐shifted shoulder at 2000–2040 cm^−1^ (insets Figure 3), whereas at 25 mbar this peak could not be distinguished. Carbonyls are generally considered to be the key intermediate species for CO_2_ methanation.^[25]^ The recent work of Mansour and Iglesia emphasizes the pivotal role of carbonyls in methanation of both CO and CO_2_.^[27]^ In the next section we will focus on the evolution of surface carbonyls and the involvement of the Co‐CeO_2_ interface in the activation of CO_2_.

### Probing Oxidation, Carburization, and Hydridization of Co/CeO_2_

**Figure 4:**
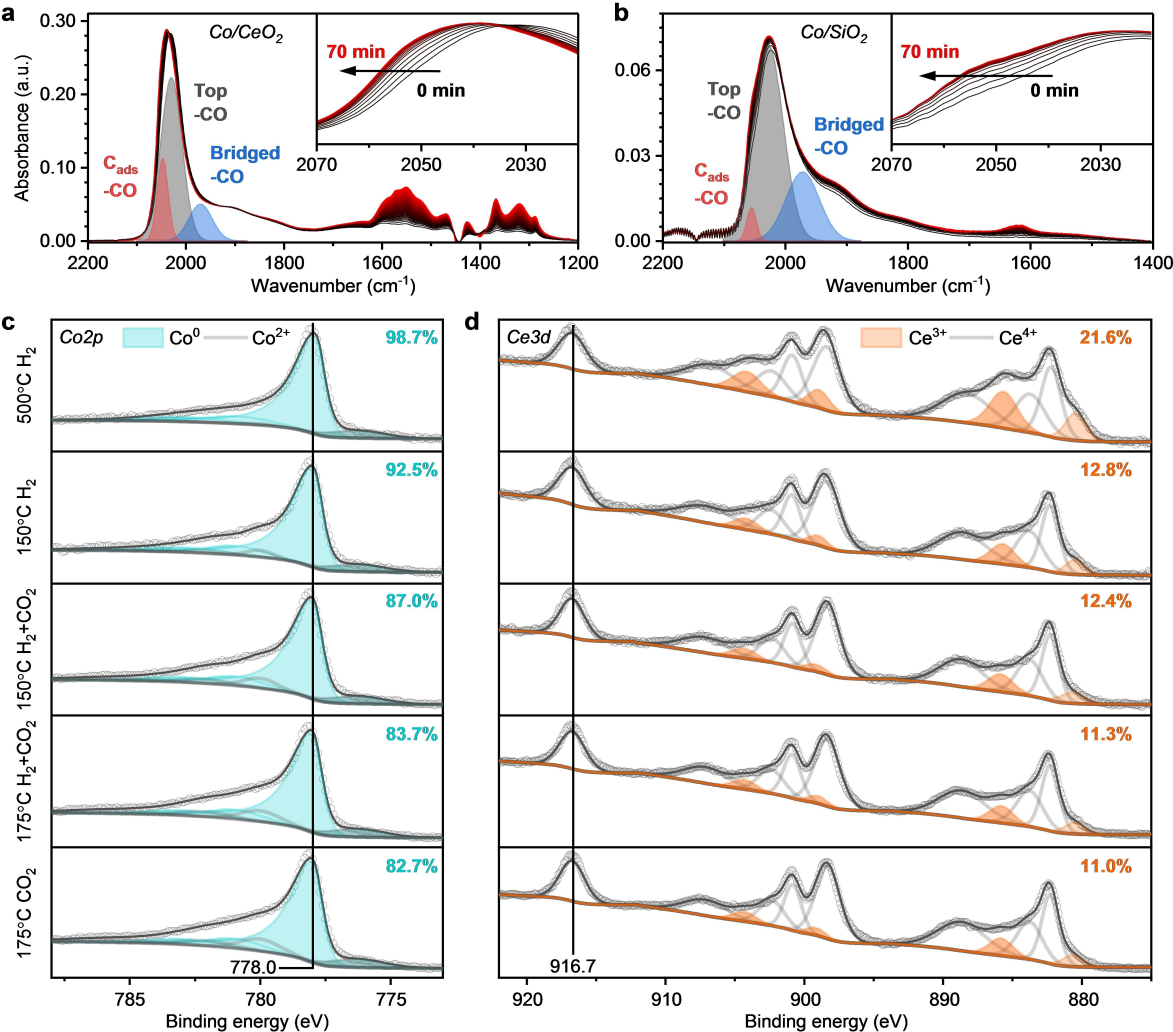
Surface behavior of Co/CeO_2_ and Co/SiO_2_. a), b) Time‐dependent CO adsorption FTIR experiment over the course of 70 min after dosing 0.5 mbar CO (at *t*=0 min) on Co/CeO_2_ (a) and Co/SiO_2_ (b). The deconvoluted peaks for the blue‐shifted CO species (C_ads_‐CO), top‐CO and bridged‐CO at *t*=70 min are shown. A full description and spectra for 0.1 mbar can be found in Note S2. The absorbance for each spectrum was background corrected and normalized by pellet weight. Conditions: 50 °C, 4 h reduction in H_2_ at 500 °C. c), d) In situ lab‐based NAP‐XPS study of Co/CeO_2_. c) Co 2*p* region (grey area corresponds to Co LMM Auger contributions). d) Ce 3*d* region. The top row was acquired after reduction at 500 °C in H_2_. The catalyst was subsequently cooled down in hydrogen and exposed to different temperatures and gas compositions.

To investigate the nature of the carbonyl species, CO‐FTIR experiments were performed at 50 °C on reduced Co/SiO_2_ and Co/CeO_2_ samples. Based on time‐ and pressure‐dependent CO adsorption experiments (Figure 4a,b, Note S2, and Figures S8–S13), the appearance of the blue‐shifted carbonyl band around 2047 and 2057 cm^−1^ for Co/CeO_2_ and Co/SiO_2_, respectively, can be explained by the adsorption of CO on sites adjacent to C_ads_ or O_ads_ atoms formed during CO dissociation.[^23^, ^28^] The presence of C_ads_ and O_ads_ on the surface is corroborated by the evolution of gaseous CO_2_ (formed via the Boudouard reaction) over both catalysts. In situ formation of CO_2_ also led to the appearance of carbonate, bicarbonate, and formate bands (1650–1200 cm^−1^, Figure 4a) on Co/CeO_2_.[^3^, ^24^] An alternative explanation for the observed blue‐shifted carbonyl band is a partial oxidation of the cobalt surface by adsorbed oxygen atoms (O_ads_).[^21^, ^29^] We note that both explanations are in line with the time‐dependent evolution of the additional carbonyl band upon exposure to 0.5 mbar of CO (Figure 4a,b), as the adsorbed species are formed during the exposure to CO.

The possibility of the partial surface oxidation of Co/CeO_2_ by O_ads_ was investigated by in situ NAP‐XPS experiments. To follow the oxidation state of cobalt and ceria upon exposure to CO, the percentage of Co^0^ and Ce^3+^ were determined from the Co 2*p* and Ce 3*d* core line spectra, respectively. After treatment in hydrogen (1 mbar) at 500 °C, the Co_3_O_4_ precursor was completely reduced (Figure S14–S15). Additionally, the concentration of reduced Ce^3+^ species increased by ≈20 %, in line with the hydrogen temperature‐programmed reduction results (Figure S16). Following the procedure of the FTIR experiment, the cell was evacuated to a high vacuum (10^−8^ mbar) at 500 °C and then cooled down to 50 °C. Upon exposing the sample to CO, no significant oxidation of cobalt was observed. Although lab‐based NAP‐XPS does not provide the utmost surface sensitivity (inelastic mean free path of Co 2*p* photoelectrons is ≈15 Å), we infer that these results disfavor the hypothesis of the partial oxidation of the cobalt surface by O_ads_. The observed slight oxidation of Ce^3+^ (18.7 % to 16.8 % Ce^3+^) under the same conditions (Figure S14b), however, indicates the dissociation of CO. In this process, C_ads_ remains on the Co surface while O_ads_ oxidizes the CeO_2_ support via oxygen spillover or reacts with *CO to form CO_2_ as observed by FTIR (Note S2).^[8]^ Thus, the additional carbonyl peak in FTIR around 2047 cm^−1^ (subsequently referred to as C_ads_‐CO) is likely caused by the carburization of the cobalt surface.

Next, the redox behavior of cobalt and ceria under reaction conditions was investigated by exposing the reduced catalyst to a reaction mixture with 0.2 mbar CO_2_ and 0.8 mbar H_2_ at 150 °C and 175 °C (Figure 4c,d). After pretreatment, cobalt was fully reduced (top row Figure 4c) and ceria was substantially reduced (21.6 % Ce^3+^, top row Figure 4d). Instead of cooling down in vacuum, as in the previous experiment, the sample was cooled down in hydrogen to 150 °C as during the activity tests. Counterintuitively, the presence of hydrogen during cooling resulted in a significant decrease of the Ce^3+^ concentration to 12.8 %. The apparent oxidation of Ce^3+^ to Ce^4+^ can be explained by the formation of cerium hydride species (Ce^4+^H^−^, Note S3).[^30^, ^31^] Consistent with these results, the oxidation of Ce^3+^ continued during further cooling to 50 °C in the presence of H_2_ (9.3 % Ce^3+^, Figure S18). Oxidation of cobalt (+6.2 % Co^2+^) observed by NAP‐XPS upon cooling to 150 °C in H_2_ (Figure 4c) can be linked to a reverse oxygen spillover at the cobalt‐ceria interface, pointing to the strong interaction between cobalt and ceria.[^8^, ^32^] Exposing the catalyst to reaction conditions (H_2_+CO_2_) leads to a gradual oxidation of the Co particles (Figure S19). At 150 °C we observed 87.0 % of Co^0^ which decreased to 83.7 % of Co^0^ at 175 °C (Figure 4c). Removing H_2_ from the reaction mixture at 175 °C resulted only in a minimal further oxidation (82.7 % Co^0^). Under reaction conditions ceria undergoes slight oxidation (Figure 4d) due to the filling of oxygen vacancies.[^8^, ^33^]

Altogether, we conclude that both Co and CeO_2_ participate in the activation of CO_2_. Activation of CO_2_ at the Co‐CeO_2_ interface under reaction conditions involves time‐ and temperature‐dependent partial oxidation of both Ce^3+^ and Co^0^. With this better understanding of the active site speciation, we focus in on operando FTIR spectroscopy to monitor the reaction intermediates.

### Steady‐State Operando FTIR Spectroscopy

**Figure 5:**
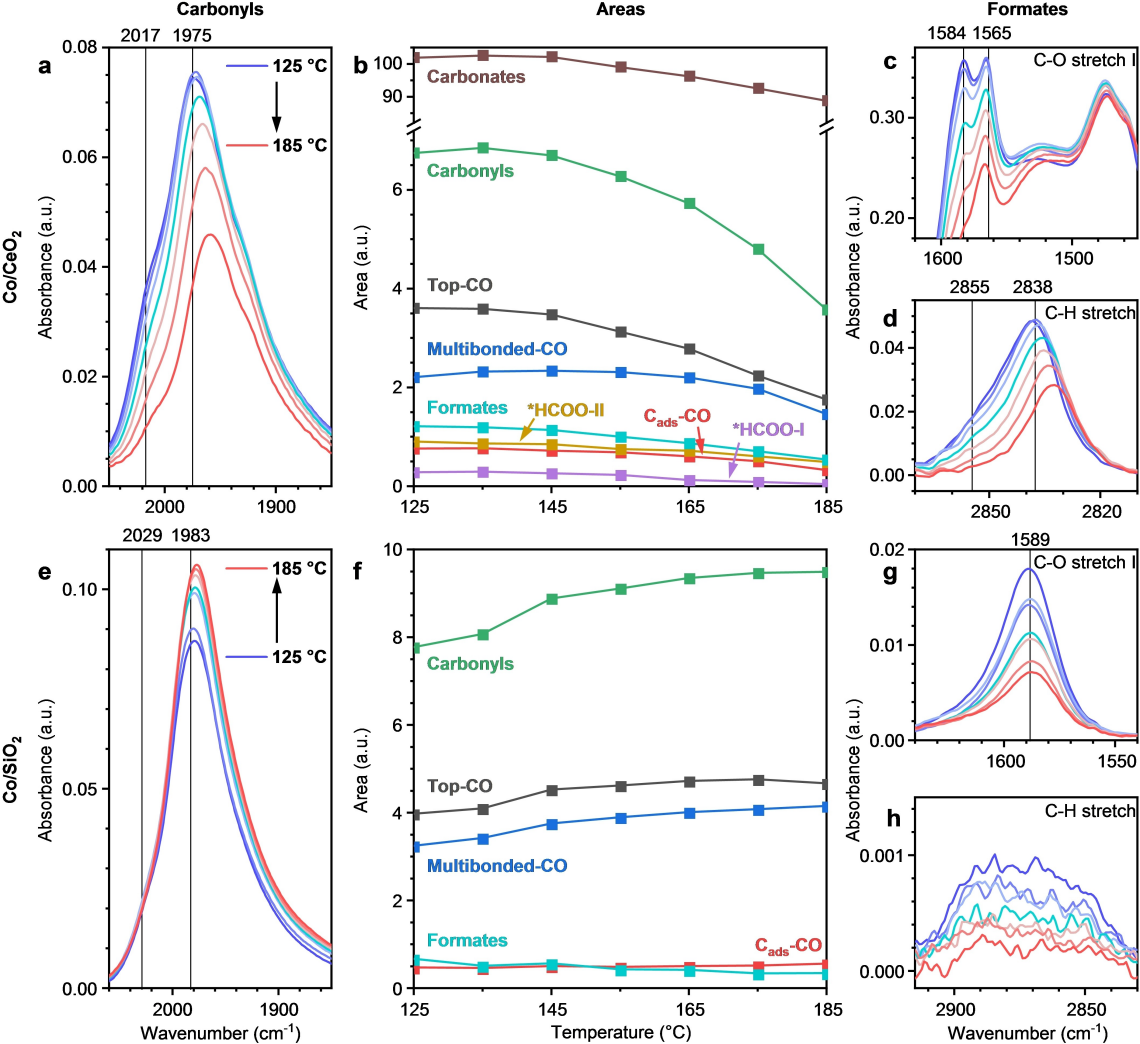
Temperature‐resolved operando FTIR for Co/CeO_2_ (a–d) and Co/SiO_2_ (e–h) under steady‐state conditions at 0.6 mbar of CO_2_. a), e) Carbonyl region including the positions of C_ads_‐CO and top‐CO at 125 °C. b), f) Peak area evolution over temperature obtained by deconvolution of the spectra. For the formates, the C−O stretch‐I area is used. c), g) Formate C−O stretch region. d), h) Formate C−H stretch region. The lines indicate the positions of the formate species at 125 °C. Entire scans are provided in Figures S20 (Co/CeO_2_) and S21 (Co/SiO_2_). All spectra and areas are normalized by pellet weight. Conditions: 0.6 mbar CO_2_ and 200 mbar H_2_ in He, total flow 200 mL min^−1^, 125–185 °C.

Operando FTIR spectroscopy experiments were performed between 125 and 185 °C with a CO_2_ partial pressure of 0.6 mbar to investigate the influence of temperature on the steady‐state behavior of various surface species (Figure 5). The area of carbonyl signal strongly decreased between 125 and 185 °C on Co/CeO_2_ (Figure 5a). The decrease in the total amount of carbonyls can primarily be ascribed to the decrease in linear carbonyls (Figure 5b). For top‐CO, a significant red shift from 1975 to 1963 cm^−1^ and decrease in the peak area were observed from 145 °C onwards. The red shift is most likely caused by the decrease in *CO coverage and thereby a decrease in lateral carbonyl‐carbonyl interactions, as observed in previous CO adsorption experiments (Note S2).^[21]^ In contrast to linear carbonyls, multibonded carbonyls were more stable over the investigated temperature range—their quantity started decreasing significantly only above 165 °C. We note that Meunier and co‐workers demonstrated that for CO hydrogenation on a Co/Al_2_O_3_ catalyst these multibonded carbonyls were the main reaction intermediates under the applied reaction conditions.^[34]^

The ceria‐based catalyst contained two types of formates, one with a C−O stretch I vibration band at 1584 cm^−1^ and one at 1565 cm^−1^ (Figure 5c). In the C−H stretching vibration region, two contributions were observed as well (≈2855 and ≈2838 cm^−1^, Figure 5d).^[24]^ Moreover, two bands at 1369 and 1359 cm^−1^ were assigned to the C−O stretch II vibration. Using 2D correlation spectroscopy and H/D exchange experiments (Note S4 and Figures S22–S25), we found that peaks located at 2855, 1584, and 1369 cm^−1^ correspond to one formate species (formate‐I) and peaks located at 2838, 1565, and 1359 cm^−1^ to another formate species (formate‐II). Two types of formates were also reported previously for Ru/Al_2_O_3_,^[35]^ Ru/TiO_2_,^[36]^ and Co/silica‐alumina catalysts.^[23]^ The total amount of formates decreased steadily from 145 °C to 185 °C (Figure 5b). Formate‐I was only present to a minor extent at higher temperatures. The intensity of the carbonate bands, present in the same wavenumber range as the formate C‐O stretch bands, strongly decreased above 145 °C.

In striking contrast to Co/CeO_2_, the area of the carbonyl peaks on previously reported catalysts and Co/SiO_2_ (Figure 5e) increased within the studied temperature range.[^3^, ^15^, ^22^, ^25^, ^37^] Deconvolution showed that the intensity of C_ads_‐CO remained approximately constant, while the other carbonyls contribute to the increase of the total peak area (Figure 5f). The total carbonyl amount increased by ≈20 % from 125 °C to 185 °C. Over the same temperature range, the area of the carbonyl peaks decreased by ≈50 % on Co/CeO_2_. In line with the catalytic data, the different trend demonstrates that Co/CeO_2_ is active for CO_2_ dissociation at a much lower temperature compared to other catalysts. We should note that on Co/SiO_2_ the position of the carbonyl band was found at higher wavenumbers than on Co/CeO_2_ at all temperatures. The higher wavenumbers might indicate a weaker bonding of the carbonyls to Co/SiO_2_.^[38]^ The lower bonding strength can contribute to the differences in the observed catalytic performance. For instance, the lower bonding strength can lead to the higher CO selectivity of Co/SiO_2_ (Figure S5).^[39]^

Similar to Co/CeO_2_, the intensity of the formate bands on Co/SiO_2_ decreased over temperature (Figure 5g,h). Wang et al. also reported a decrease in formate intensity on Pd/Al_2_O_3_ at an increasing temperature.^[25]^ The decreasing area of the formate peaks in combination with the increasing carbonyl peaks over temperature on Co/SiO_2_ might indicate that the formates decompose into carbonyls in the catalytic cycle.^[25]^ In this scheme, the rate of formate decomposition increases with temperature, whereas the conversion of *CO to CH_4_ is still slow. An alternative possibility is that formates do not play an important role in the mechanism and remain mere spectators.^[40]^ To clarify the catalytic role of the surface intermediates and to further elucidate the properties of the different formate species on Co/CeO_2_, next we performed operando FTIR under transient conditions.

### Transient Operando FTIR Spectroscopy

**Figure 6:**
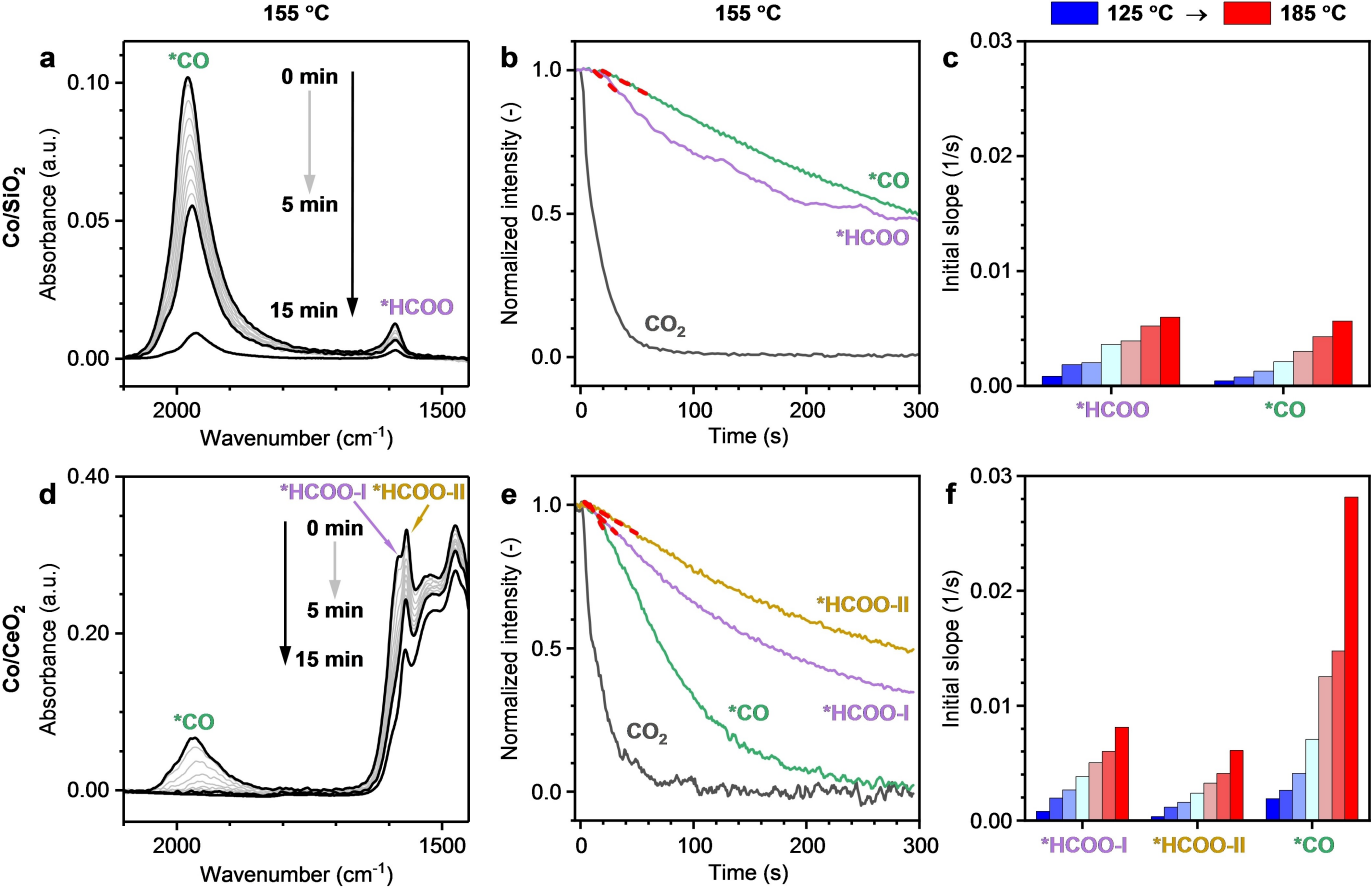
Operando transient kinetic step response FTIR on a)–c) Co/SiO_2_ and d)–f) Co/CeO_2_. a), d) CO_2_/H_2_/He to H_2_/He switch at 155 °C. Black bold lines indicate scans acquired at 0, 5, and 15 minutes. Gray lines show the decay in species in the first 5 minutes. Carbonyls are labeled by *CO; formates by *HCOO. b), e) Normalized switch of Figure 6a and d, respectively, for carbonyls and formates at 155 °C. Gaseous CO_2_ (black) is given for comparison. Fitted initial slopes are indicated by red dashed lines. c), f) Initial slopes of the normalized decays for Co/SiO_2_ and Co/CeO_2_, respectively. Conditions: 0.6 mbar CO_2_ and 200 mbar H_2_ in He, total flow 200 mL min^−1^, 125–185 °C.

Spectroscopic analysis of surface coverages during transient kinetic experiments is a powerful tool to reveal the mechanistic peculiarities of catalytic reactions.^[41]^ In the transient kinetic step‐response experiments of this work, the feed flow was rapidly switched from CO_2_/H_2_/He to H_2_/He while the response of the adsorbed species was monitored by acquiring time‐resolved FTIR spectra. The intensity of all bands on both catalysts decreased after the switch (Figure 6). For Co/SiO_2_, the intensity decays fast in the first five minutes (gray lines in Figure 6a) and after that the decay slows down. After 15 minutes, a small fraction of both the formates and carbonyls (23 % and 9 %, respectively, at 155 °C) was still present on the Co/SiO_2_ surface. The reaction temperature governs the response of the surface species. For example, at 125 °C a large fraction of the carbonyls (66 %) and formates (55 %) was not removed from Co/SiO_2_ after 15 minutes (Figure S26a–c). In contrast, almost no carbonyls and formates remained on the surface after 15 minutes at 185 °C. Both carbonyl and formate species are significantly more dynamic on Co/CeO_2_ and decompose faster at all temperatures.

To compare the transient behavior of different species at different temperatures, the band intensities were normalized (Figure 6b). Herein, the absorbance of the species of interest under steady‐state reaction conditions is taken as unity and the absorbance before the feed was switched as zero. The time scale at which the formates and carbonyls are decomposed or hydrogenated is similar (Figure 6b), in line with a methanation mechanism involving both species.^[25]^ The formate species on Co/SiO_2_ display a fast initial decomposition rate followed by a period of slower decomposition (Figure 6b and Figure S27). A possible explanation for the fast and slow components could be the presence of different unresolved formate species on Co/SiO_2_ (akin to the two formates observed on Co/CeO_2_).[^35^, ^36^, ^42^] A certain degree of peak asymmetry observed for the formate C−O (1589 cm^−1^) and C−H (2830–2910 cm^−1^) stretch vibrations on Co/SiO_2_ supports this explanation (Figure 6a and Figure 5g,h). Other possible explanations are a change of the catalyst surface related to varying surface coverages or a slight reduction of the cobalt surface after the switch to pure hydrogen.[^21^, ^23^] The apparent decomposition rates were determined by fitting slopes to the normalized decay just after the feed was rapidly switched (indicated in red in Figure 6b,e). In this manner, the surface coverages are still close to the coverages at steady‐state (>90 %). Thus, the initial rates provide qualitative information about the hydrogenation/decomposition rates of the species. The initial rate of disappearance of the formates is faster than that of the carbonyls for all switches over Co/SiO_2_ (Figure 6c). The faster removal of the formates suggests that the formates precede the carbonyls in the reaction pathway.^[25]^ To rule out a change in surface species coverages or surface changes as the main cause for the faster initial decomposition of formates compared to carbonyls on Co/SiO_2_, a steady‐state isotopic transient kinetic analysis (SSITKA) FTIR experiment was performed. In SSITKA, a switch between chemically identical feeds containing ^12^CO_2_ and ^13^CO_2_ isotopologues is made. Thus, in SSITKA‐FTIR all spectra are acquired under steady‐state. SSITKA‐FTIR results at 175 °C (Figure S28) demonstrated that the isotope exchange of formates precedes that of carbonyls, in line with the observations based on the transient kinetic step‐responses. The step‐response experiments on Co/CeO_2_ demonstrate a faster decomposition of the carbonyls compared to Co/SiO_2_. At 155 °C, all the carbonyls were removed after 5 minutes on Co/CeO_2,_ while for Co/SiO_2_ only 50 % of them were removed (Figure 6d,e). Already at 125 °C, approximately 50 % of the carbonyls were removed after 5 minutes from Co/CeO_2_ (only 10 % for Co/SiO_2_) (Figure S26d–f). It should be noted that the fast removal of carbonyls from Co/CeO_2_ is not caused by their desorption as demonstrated by a control experiment in which the feed was rapidly changed to He instead of H_2_/He (Figure S29). The initial rate of carbonyl conversion was up to 5 times higher on the ceria‐supported catalyst (Figure 6f). As the carbonyl hydrogenation is slower than the decomposition of formates to carbonyls, the observed higher apparent rate of carbonyl hydrogenation on Co/CeO_2_ defines the higher catalytic activity of this material.

The fast dynamics of both formates on Co/CeO_2_ suggests that these species are active reaction intermediates. The step‐response and SSITKA‐FTIR experiments highlighted a difference in the transient behavior of the two different formates (Figure 6e and Figure S30). Formate‐I exchanged faster than the red‐shifted formate‐II (Figure 6f). 2D correlation analysis confirmed this conclusion (Note S4). Therefore, formate‐I is most likely the active formate in the dominant reaction pathway. Although the difference in nature of the two formates is not yet understood, it is hypothesized that these species are bound on different surface sites, e.g. the more active formate‐I is at the Co‐CeO_2_ interface and formate‐II on CeO_2_, as previously proposed.[^23^, ^35^, ^36^, ^42^]

Comparing formate‐I on Co/CeO_2_ to the formate species on Co/SiO_2_, a faster transient behavior on Co/CeO_2_ is observed. At a low temperature, formate‐II is less dynamic than the formate on Co/SiO_2_. However, upon increasing the temperature to 185 °C this formate also decomposes faster than the formate on Co/SiO_2_. Given the relatively large quantity of the formate‐II species on Co/CeO_2_ (Figure 5b), they can also contribute to the higher activity on Co/CeO_2_, especially at higher temperatures. The reaction orders in CO_2_ with respect to CH_4_ at 175 °C for Co/CeO_2_ (Figure S31) are consistent with the effciient conversion of CO_2_ to *CO via *HCOO on Co/CeO_2_. In contrast to Co/SiO_2_, for which a shortage of reactive carbon species on the catalyst surface can be inferred from positive orders in CO_2_, the orders for Co/CeO_2_ are close to zero or even slightly negative (Figure S31). Carbon‐containing species formed upon adsorption and activation of CO_2_ on ceria (e.g., the observed carbonates and bicarbonates, absent on silica) can act as precursors to the reactive formates and carbonyls. This point is supported by step‐response experiments at a higher partial pressure of CO_2_ (25 mbar, Note S5 and Figure S32). The increased partial pressure of CO_2_ leads to a significantly increased response time of the carbonyl and formate bands on Co/CeO_2_, whereas for Co/SiO_2_ the response was almost identical. Moreover, for Co/CeO_2_ a sustained methane production was observed by mass spectrometry for ≈50 s after the switch to H_2_, which was not observed for the Co/SiO_2_ catalyst, lacking the adsorbed carbon‐containing species necessary to sustain methane formation (Figures S6 and S33). In summary, CeO_2_ provided a sufficient pool of active formates that on SiO_2_ was much smaller. Moreover, significantly faster dynamics of carbonyls and formates on Co/CeO_2_ compared to Co/SiO_2_ explain the higher activity of the ceria‐supported catalyst.

## Conclusion

In this work, we demonstrated that Co/CeO_2_ displays a superior low‐temperature (<200 °C) CO_2_ methanation activity and selectivity at CO_2_ partial pressures down to the current concentration of CO_2_ in the atmosphere—0.4 mbar. The mechanistic origin of the unusual activity of Co/CeO_2_ was studied by detailed spectroscopic investigations and compared to a conventional Co/SiO_2_ catalyst. Reaction order studies and in situ NAP‐XPS, point at the importance of the CeO_2_ support and Co‐CeO_2_ interface in adsorbing and activating CO_2_. In addition, our NAP‐XPS data showcased the possibility of hydride formation on CeO_2_ with deposited cobalt nanoparticles. The combination of steady‐state, transient step‐response, and SSITKA‐FTIR experiments allowed for the identification of formates and carbonyls as the key reaction intermediates. Furthermore, an additional linear carbonyl species (C_ads_‐CO) was observed and, using 2D correlation analysis, linked to the facile CO dissociation on the catalyst surface. This C_ads_‐CO species can be used as a proxy for CO dissociation in future mechanistic studies. Our results point at a mechanism in which CO_2_ is activated on the support and at the metal‐support interface, leading to formates that further decompose into carbonyls. These carbonyls are subsequently hydrogenated to methane on the Co surface. We should note that we do not rule out a contribution of direct CO_2_ dissociation at Co‐CeO_2_ interface to form carbonyls.^[8]^ Hydrogen dissociation on the Co surface also leads to hydrogen spillover to the Co‐ceria interface and ceria support to form formates and restore oxygen vacancies. Both the decomposition of formates to carbonyls and the hydrogenation of carbonyls to CH_4_ were significantly faster on Co/CeO_2_ than on Co/SiO_2_. Moreover, the formate pool was much larger for the CeO_2_‐based catalyst. The combination of a higher concentration of active intermediates and the higher intrinsic activity of formates and carbonyls explains the high activity of Co/CeO_2_ at low partial CO_2_ pressures and low temperatures. These results emphasize the role of the metal‐ceria interface in CO_2_ hydrogenation chemistry and provide a foundation for the development of better catalysts for the utilization of CO_2_.

## Conflict of interest

The authors declare no conflict of interest.

1

## Supporting information

As a service to our authors and readers, this journal provides supporting information supplied by the authors. Such materials are peer reviewed and may be re‐organized for online delivery, but are not copy‐edited or typeset. Technical support issues arising from supporting information (other than missing files) should be addressed to the authors.

Supporting Information

## Data Availability

The data that support the findings of this study are available from the corresponding author upon reasonable request.
