# Supplementary material for: Ceria‐Supported Cobalt Catalyst for Low‐Temperature Methanation at Low Partial Pressures of CO2
Source: Angew Chem Int Ed Engl. 2022 Dec 22;62(5):e202214864. doi: 10.1002/anie.202214864 (PMC10107782; doi:10.1002/anie.202214864)
Supplement: Supplementary file 1 — Supporting Information [file ANIE-62-0-s001.pdf]

Angewandte Chemie
Eine Zeitschrift der Gesellschaft Deutscher Chemiker

Supporting Information

Ceria-Supported Cobalt Catalyst for Low-Temperature Methanation at Low Partial Pressures of CO_2

J. J. C. Struijs, V. Muravev, M. A. Verheijen, E. J. M. Hensen, N. Kosinov**

Supplementary information

Table of Contents

Experimental Section 2
Introduction 6
Results and Discussion 13
The Ceria-Supported Cobalt Catalyst Outperforms the Silica-Supported Cobalt Catalyst 13
Operando FTIR Spectroscopy at Low and High Partial Pressures of CO_2..15
Note S1. Pressure-Dependency of Surface Coverages 16
Probing Oxidation, Carburization and Hydridization of Co/CeO_2 17
Note S2. Pressure- and Time-Dependent CO Adsorption Experiments 17
Note S3. Cerium Hydride Species 24
Steady-State Operando FTIR Spectroscopy 26
Note S4. 2D Correlation Assisted Determination of Formate Species on CeO_2..........................27
Transient Operando FTIR Spectroscopy...30
Note S5. Transient operando FTIR at different CO_2 partial pressures 33
References 35

Experimental Section

Catalyst preparation. All chemicals were purchased from Sigma-Aldrich and used without further purification. The CeO_2 support was prepared by the method of precipitation from a $Ce(NO_3)_3 \cdot 6H_2O$ precursor. First, a 0.1M aqueous solution of the ceria salt was prepared, which was subsequently precipitated by a dropwise addition of a 30% aqueous ammonia solution upon stirring until a pH of 9 was reached. After stirring the solution at room temperature for 24 hours, the precipitate was centrifuged out of the solution, washed, and dried overnight in a convection oven at 110 °C. Afterward, the obtained solid material was calcined in air by raising the temperature from room temperature to 500 °C over 8 hours and sustaining it at 500 °C for 4 hours. A commercial SiO_2 support material was used (X-080, Shell). The Co-based catalysts were prepared from these supports using a wet impregnation method with an ammonia complex[1]. To make the complex, $Co(CH_3COO)_2 \cdot 4H_2O$ was dissolved in 20 ml of deionized water and ammonia solution was added under stirring until a 20-fold molar excess of ammonia with respect to Co precursor was reached. The obtained solution was mixed with an appropriate amount of CeO_2 to obtain 10 wt% Co loading and another 20 ml of deionized water. Co/SiO_2 was prepared by the same procedure but instead of water a 1:1 H_2O:EtOH mixture was used during impregnation. The solution was then stirred at room temperature for ~30 minutes and the water was removed by evaporation. All catalysts were dried overnight at 110 °C. Lastly, a calcination at 350 °C (2 °C/min) for 4 hours was performed in a 200 ml/min flow of 20 vol% O_2 in He.

Catalytic activity. The catalytic activity of the catalysts in CO_2 methanation was assessed using a microflow reactor consisting of 10 parallel quartz tubes. A pelletized catalyst with a size between 125 and 250 µm was obtained by pressing, crushing, and sieving the catalyst material. An amount of 2.5, 5, 10, 20, and 50 mg for Co/CeO_2 and 10, 20, 50, and 100 mg for Co/SiO_2 of the obtained catalyst was subsequently well mixed with SiC of the same sieve fraction. The diluted samples were then loaded into the reactor tubes (i.d. 4 mm) placed between two quartz wool plugs. An equal pressure drop was ensured for each tube by the addition of quartz wool. The catalysts were reduced in situ after placing the quartz tubes into the reactor. The reduction procedure was performed in 20 vol% H_2 in He and consisted of controlled heating of 10 °C/min to 500 °C followed by a 4 h isothermal dwell and cooling down to the desired reaction temperature. All experiments, including reduction, were performed at atmospheric pressure with a total flow rate of 50 ml/min STP per reactor.

The influence of the reaction temperature on the catalytic activity was determined in a reaction mixture of 0.4 mbar CO_2 and 20 vol% H_2 in He. In these tests, temperatures ranging from 110 to 200 °C were measured with 10 °C increments. The temperature was increased between measurements with a rate of 5 °C/min. The reaction was stabilized at the new temperature for 20 min. After that, the products of the reaction were evaluated for 75 minutes. Measurements with different catalyst loadings (with CO_2 conversions <30%) were averaged and error bars show the standard deviation between these measurements. To determine the reaction order in CO_2, the CO_2 partial pressure was varied between 0.4 and 50 mbar at 175 °C. For all measurements a total flow of 50 ml/min STP per reactor with 20 vol% of H_2 in He was ensured by varying the He flow. After the reaction was stabilized (at least 10 min), the effluent gasses were analyzed for 75 minutes. Three GC measurements (CO_2 conversions <10%, except 2.5 mg Co/CeO_2 at 400 ppm for which the conversion was 15.0%) were averaged and error bars show the standard deviation between these injections.

The effluent gasses of the reactors were analyzed using a mass spectrometer (ThermoStar GSD 320 T2) and online gas chromatograph (Interscience CompactGC) equipped with Restek Rt-Q-Bond and Rt-Msieve 5 Å (TCD), Restek Rt-U-Bond and Rt-Q-Bond (TCD), and Restek Rtx-1 (FID) columns. In order to obtain accurate CO_2 concentrations, the flowrates of the inlet gasses were carefully controlled and the inlet CO_2 concentration was checked by GC as well. To achieve higher precision for CO_2 partial pressures below 5 mbar, the inlet CO_2 concentration was also verified by an internal argon standard that was present in the CO_2 feed.

Characterization. Inductively coupled plasma optical emission spectrometry (ICP-OES) with a charge-coupled device (CCD) spectrometer was used to determine the exact metal content. 25 mg of catalyst sample was dissolved in a mixture of acids depending on the support and subsequently diluted. Silica-supported samples were dissolved in a 1:1:1 ratio by volume of HF:HNO_3:H_2O. For CeO_2 supported catalysts, concentrated H_2SO_4 was used.

For a typical hydrogen temperature-programmed reduction (H_2-TPR) experiment, 100 mg of the studied catalyst was loaded between two layers of quartz wool in a quartz U-tube reactor. After the reactor was placed in the Micromeritics Autochem II 2920 instrument, the sample was pretreated in 5 vol% O_2 in He by 5 °C/min heating to 350 °C followed by a 35 °C/min cooling down. Subsequently, the reduction was performed by heating the reactor with 10 °C/min to 600 °C from room temperature in a 4 vol% H_2 in N_2 mixture with a total flow rate of 50 mL/min. During the reduction, the H_2 consumption was recorded by a thermal conductivity detector (TCD).

CO chemisorption measurements were performed on reduced samples using a Micromeritics ASAP 2010C instrument. To reduce the as-prepared samples, the samples were dried in vacuum at 110 °C and subsequently heated in flowing H_2 with a rate of 10 °C min^{-1} to 500 °C. At the final reduction temperature, the sample was reduced for 4h. Lastly, the samples were evacuated at 520 °C for 60 min. The CO adsorption isotherms were measured at 30 °C. An extrapolation of the linear part of the isotherm was used to determine the CO/Co ratios at zero pressure. For the particle size estimations, a hemispherical geometry and a CO/$Co_{surface}$ adsorption stoichiometry of 1.5 were assumed.

TEM measurements were performed on a JEOL ARM 200F Transmission Electron Microscope operated at 200 kV equipped with a 100 mm^2 Centurio SDD EDX detector. Samples were prepared by dropping 4 drops of a suspension of reduced and passivated catalysts on a holey carbon film and allowing it to dry at ambient conditions.

Operando FTIR. Operando Fourier-transform infrared spectroscopy experiments were performed using a homemade operando transmission IR cell in a Bruker Vertex 70v FTIR spectrometer equipped with a deuterated-triglycine sulfate detector. The catalysts were pressed into thin wafers (13 mm in diameter) obtained by pressing around 5–15 mg of sample. Before measurements, the catalyst was reduced in situ at 500 °C (10 °C/min) in a 20 vol% hydrogen mixture in He with a total flow of 200 ml/min for 4 hours and subsequently cooled down to the temperature of the experiment. At all times, the effluent gas was analyzed by a ThermoStar GSD 320 T2 mass spectrometer to monitor the products formed in the reaction ($^{13}CO_2$ (m/z = 45), $^{12}CO_2$ (m/z = 44), ^{13}CO (m/z = 29), ^{12}CO (m/z = 28), $^{13}CH_4$ (m/z = 17), $^{12}CH_4$ (m/z = 15)).

Each spectrum was obtained by recording and averaging 32 scans in the 4000-900 cm^{-1} range with a resolution of 2 cm^{-1}. To follow the transient behavior when switching between H_2/He and CO_2/H_2/He mixtures (transient kinetic step response) or when switching between $^{12}CO_2$/H_2/He and $^{13}CO_2$/H_2/He

mixtures (steady-state isotope transient kinetic analysis – SSITKA), the spectra were rapidly taken after each other. These spectra also had a resolution of 2 cm^{-1} and were taken from 4000 to 900 cm^{-1}, but with a lower number of averaged scans (typically 2 to 6 scans depending on the time resolution needed for the transient behavior, with less averaging for faster transients).

All SSITKA experiments were performed at 175 °C with a 200 ml/min flow (STP) of 25 mbar CO_2 and 20 vol% H_2 in He. Step responses were measured with 0.6 mbar CO_2 (125-185 °C, 10 °C steps) and 25 mbar CO_2 (175 °C and 200 °C), both in a 20 vol% H_2 in He flow of 200 ml/min at STP. Additionally, CO and CO_2 adsorption experiments were performed at 50 °C. Preceding these experiments, the samples were reduced at 500 °C in hydrogen. However, in contrast to the transient experiments, the cell was evacuated to a high vacuum (order of 10^{-6} mbar) at 500 °C to remove any remaining surface species. After 30 min evacuating at 500 °C, the sample was cooled down to 50 °C, after which the evacuation was stopped. For the adsorption experiments, repeatedly a small amount of CO or CO_2 was dosed to the cell in a controlled way followed by an FTIR scan. This procedure was repeated until a pressure of 10 mbar was reached.

The FTIR results were processed using a self-made MATLAB script. First, the background was corrected with respect to the scan taken after reduction or before the experiment was started. Additionally, a vertical correction followed by a correction of the slope of the baseline was performed. Spectra usually were normalized for pellet weight, as indicated in the respective figure captions. Deconvolution was performed by fitting several Gaussian contributions on a region of interest in the corrected spectra. The position of the peak was allowed to shift within a certain window as was also the case for the height and standard deviation of the peak. The standard deviation (σ) described the width of the peak. The relation between the full width half maximum (FWHM) and the standard deviation can be expressed as $FWHM = 2\sigma\sqrt{2\ln 2} \approx 2.4\sigma$. Two-dimensional correlation analysis was processed using a MATLAB script as well. The theory of this method is extensively described in the book of Noda[2]. For a summary of the technique, the following paper of Noda is recommended[3].

In situ NAP-XPS. In situ NAP-XPS was carried out on a SPECS system. The core-line spectra were obtained using monochromatized Al Kα irradiation (1,486.6 eV) generated by an Al anode (SPECS XR-50) and an excitation source power of 50 W. The electron analyzer (SPECS Phoibos NAP-150) allows for normal-emission XPS measurements in the presence of gases (up to ∼20 mbar) owing to the differential pumping system. A continuous gas flow (2 or 3 ml/min STP) was supplied to the cell by calibrated mass-flow controllers. High purity gasses (99.999%) were used. The total pressure in the NAP reaction cell was kept constant at 2 or 3 mbar using an electronic back-pressure regulator. The reduction gas mixture consisted of a 1 mL/min H_2 and 2mL/min Ar flow. After this mixture was introduced to the cell and total pressure was set to 3 mbar, the sample was heated to 500 °C and kept at 500 °C for 4h. Then, depending on the experiment, the cell was evacuated and cooled down or immediately cooled down in hydrogen to the desired temperature. Subsequently the sample was exposed to a reaction mixture at the desired temperature. For the CO exposure experiments, the feed was 2 mL/min. During CO_2+H_2 reaction experiments the feed was 0.3 mL/min CO_2, 1.2 H_2 mL/min and 1.5 Ar mL/min and the total pressure in the cell was fixed to 2 mbar. All the XPS spectra were recorded isothermally. The total acquisition time of the spectra (including a survey spectrum and O 1*s*, C 1*s*, Ce 3*d*, and Co 2$p_{3/2}$ regions) was approximately 2-3 h. A pass energy of 40 eV was typically used with a dwell time of 0.5 s and a step size of 0.1 eV. The U‴ (Ce^{4+}) component of the Ce 3*d* line with a characteristic position of 916.7 eV was used to correct the binding energies of the Co 2$p_{3/2}$ and Ce 3*d* regions[4–6]. This approach allowed for a reliable energy

calibration of the photoelectron spectra at all reaction conditions. A standard procedure involving use of Shirley background subtraction and atomic sensitivity factors was applied for data processing. Spectral lines were fitted using the CasaXPS software by a symmetric pseudo-Voigt function referred to as GL(30). Only the main metallic component of Co was fitted by the asymmetric LA(1.2,5,5) lineshape in the CasaXPS software[7]. The Ce 3*d* line was fitted according to a model described elsewhere[5,8].

Introduction

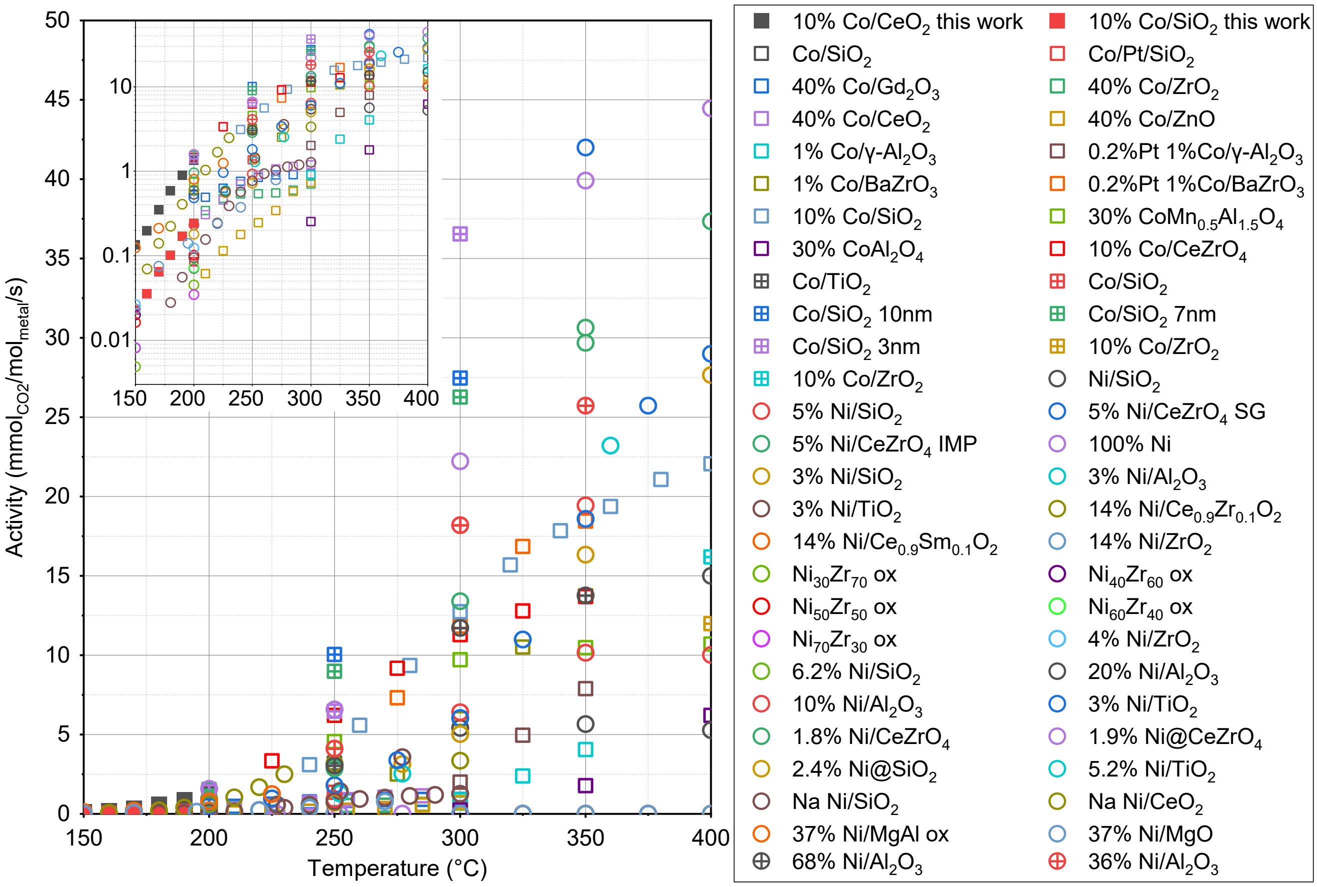

Figure S1 *Activity comparison of cobalt and nickel-based CO_2 hydrogenation catalyst at different temperatures expressed as moles of CO_2 converted per second per mole of metal present. Exact values, conditions, and references can be found in Table S1. Cobalt catalysts are denoted by ■ (atmospheric pressure with 0.4 mbar CO_2, this work), □ (atmospheric pressure) or ⊞ (elevated pressure). Ni catalysts are denoted by ○ (atmospheric pressure) or ⊕ (elevated pressure). Even at a demanding low partial pressure of CO_2 of 0.4 mbar (being significantly lower than other references shown), the Co/CeO$_2$ catalyst reported in this work outperforms the few catalysts reported at temperatures lower than 200 °C.*

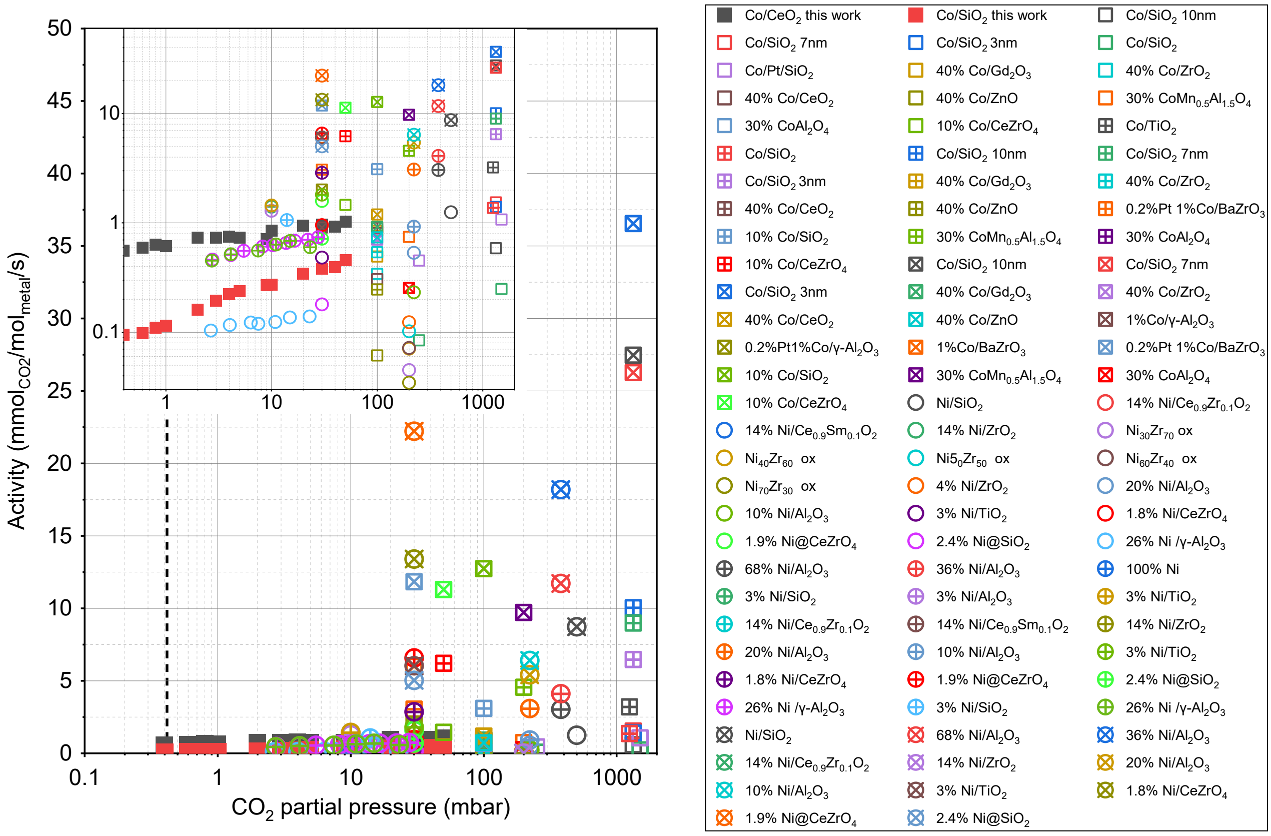

***Figure S2** Activity comparison of cobalt and nickel-based CO_2 hydrogenation catalyst at different CO_2 partial pressures expressed as the moles of CO_2 converted per second per mole of metal present. Exact values, conditions, and references can be found in Table S1. Cobalt catalysts are denoted by ▰ (175 °C, this work), ▱ (200 °C), ⊞ (250 °C) or ⊠ (300 °C). Ni catalysts are denoted by ⬭ (200 °C), ⊕ (250 °C) or ⊗ (300 °C). The current atmospheric concentration of CO_2 is indicated by a black dashed line. Even at a lower temperature of 175 °C (compared to ≥200 °C), the Co/CeO$_2$ catalyst reported in this work outperforms the few catalysts reported at partial pressures of CO_2 lower than 10 mbar.*

Table S1 *Table with corresponding values, conditions, and references of Figures S1-2. The catalytic data present in the literature was oftentimes not explicitly given in numbers or expressed in the units $mol_{CO2}/mol_{metal}/s$. In that case, values were extracted from graphs and/or converted with the information provided by the paper.*

Ref.	Catalyst	P_{CO2} (mbar)	P_{tot} (bar)	T_{reac} (°C)	Specific activity ($mmol_{CO2}$/ mol_{metal}/s)
1[9]	10CoCZ700	50	1	200	1.46
				225	3.35
				250	6.22
				275	9.19
				300	11.3
				325	12.8
				350	13.7
2[10]	Co/TiO_2	1250	5	250	3.22
	Co/SiO_2				1.37
3[11]	Co/SiO_2 10nm	1330	6	200	0.586
				250	10.1
				300	27.5
	Co/SiO_2 7 nm			200	1.54
				250	8.99
				300	26.3
	Co/SiO_2 3nm			200	1.40
				250	6.48
				300	36.6
4[12]	Co/SiO_2	250	1	200	8.46E-02
		1500	6		0.249
	$Co/Pt/SiO_2$	250	1		0.453
		1500	6		1.08
5[13]	10% Co/ZrO_2	6000	30	400	12.0
	10% Co/ Al_2O_3				16.2
6[14]	40% Co/Gd_2O_3	100	1	210	0.491
				225	0.631
				240	0.758
				255	0.845
				270	0.900
				285	0.912
				300	0.920
	40% Co/ZrO_2			210	0.342
				225	0.476
				240	0.538
				255	0.540
				270	0.555
				285	0.599
				300	0.702
	40% Co/CeO_2			210	0.306
				225	0.455
				240	0.704
				255	0.901
				270	1.06
				285	1.14

				300	1.19
	40% Co/ZnO			210	6.15E-02
				225	0.114
				240	0.178
				255	0.247
				270	0.342
				285	0.575
				300	0.737
7[15]	1%Co/γ-Al_2O_3	30	1	300	0.907
				325	2.40
				350	4.05
	0.2%Pt1%Co/γ-Al_2O_3			300	2.02
				325	4.97
				350	7.90
	1%Co/$BaZrO_3$			275	2.52
				300	6.01
				325	10.5
				350	13.7
	0.2%Pt 1%Co/$BaZrO_3$			250	3.06
				275	7.33
				300	11.8
				325	16.9
				350	18.5
8[16]	10% Co/SiO_2	100	1	240	3.11
				260	5.60
				280	9.35
				300	12.7
				320	15.7
				340	17.8
				360	19.4
				380	21.1
				400	22.1
9[17]	30% $CoMn_{0.5}Al_{1.5}O_4$	200	1	200	0.744
				250	4.56
				300	9.73
				350	10.5
				400	10.7
				450	10.6
				500	10.3
	30% $CoAl_2O_4$			300	0.254
				350	1.78
				400	6.22
				450	8.28
				500	8.51
10[18]	Ni/SiO_2	100	1	400	15.0
		500	5	200	1.25
				300	8.73
11[19]	68% Ni/Al_2O_3	380	2	250	3.05
				300	11.7
				350	13.8

	36% Ni/Al_2O_3			250	4.11
				300	18.2
				350	25.7
12[20]	5% Ni/SiO_2	160	1	350	19.4
	5% $Ni/CeZrO_4$ SG				42.0
	5% $Ni/CeZrO_4$ IMP				30.6
13[21]	100% Ni	10	1	227	1.64E-03
				252	5.03E-03
				277	1.31E-02
	3%Ni/SiO_2			227	0.559
				252	1.40
				277	3.15
	3%Ni/Al_2O_3			227	0.591
				252	1.29
				277	2.54
	3%Ni/TiO_2			227	0.573
				252	1.44
				277	3.58
14[22]	14% $Ni/Ce_{0.9}Zr_{0.1}O_2$	200	1	200	1.02E-03
				250	8.36E-03
				300	1.47E-03
				325	1.50E-03
				350	1.71E-03
				375	1.77E-03
				400	1.75E-03
	14% $Ni/Ce_{0.9}Sm_{0.1}O_2$			150	5.82E-05
				200	1.16E-03
				250	8.68E-03
				300	1.47E-02
				325	1.55E-02
				350	1.61E-02
				375	1.63E-02
				400	1.70E-02
	14% Ni/ZrO_2			200	7.61E-04
				250	9.21E-03
				300	1.47E-02
				325	1.74E-02
				350	1.92E-02
				375	2.01E-02
				400	1.99E-02
15[23]	$Ni_{30}Zr_{70}$	200	1	150	4.85E-03
				200	4.50E-02
	$Ni_{40}Zr_{60}$			150	2.00E-03
				200	7.06E-02
	$Ni_{50}Zr_{50}$			150	1.62E-02
				200	0.102
	$Ni_{60}Zr_{40}$			150	8.03E-03
				200	7.18E-02
	$Ni_{70}Zr_{30}$			150	8.14E-03
				200	3.46E-02

	4% Ni/ZrO_2			150	2.64E-02
				200	0.123
16[24]	6.2% Ni/SiO_2	170	1	450	5.29
17[25]	20% Ni/Al_2O_3	220	1	200	0.533
				250	3.08
				300	5.42
				350	5.66
				400	5.28
				450	5.08
				500	4.82
	10% Ni/Al_2O_3			200	0.232
				250	0.927
				300	6.40
				350	10.2
				400	10.0
				450	9.64
				500	9.36
18[26]	3 % Ni/TiO_2	30	1	200	0.483
				225	0.966
				250	1.81
				275	3.38
				300	6.04
				325	11.0
				350	18.6
				375	25.7
				400	29.0
19[27]	1.8% Ni/$CeZrO_4$	30	1	200	0.958
				250	2.87
				300	13.4
				350	29.7
				400	37.3
	1.9% Ni@$CeZrO_4$			200	1.59
				250	6.58
				300	22.2
				350	39.9
				400	44.5
	2.4% Ni@SiO_2			200	0.180
				250	0.718
				300	5.03
				350	16.3
				400	27.7
20[28]	5.2% Ni/TiO_2	190	1	360	23.2
21[29]	3% Ni/SiO_2	2.76	1.4	252	0.462
		4.13			0.510
		5.47			0.558
		8.26			0.608
		10.3			0.624
		13.8			0.657
		16.6			0.690
		22.1			0.704

		27.6			0.740
22[30]	3% Ni/SiO_2	14	1.4	227	0.474
				252	1.06
				277	2.79
23[31]	26% Ni/γ-Al_2O_3	2.67	1	200	0.104
		4.03			0.116
		6.38			0.123
		7.52			0.119
		10.8			0.124
		15.0			0.137
		23.0			0.140
		2.74		230	0.451
		4.12			0.517
		7.48			0.559
		11.0			0.635
		15.0			0.686
		23.1			0.599
24[32]	Na Ni/SiO_2	10	1	180	2.78E-02
				190	5.56E-02
				200	9.45E-02
				210	0.156
				220	0.245
				230	0.389
				240	0.573
				250	0.773
				260	0.934
				270	1.02
				280	1.13
				290	1.20
				300	1.27
	Na Ni/CeO_2			160	7.00E-02
				170	0.140
				180	0.224
				190	0.406
				200	0.602
				210	1.04
				220	1.68
				230	2.49
				250	3.18
				300	3.36
25[33]	37% Ni/MgAl ox	150	1	150	0.125
				170	0.211
				200	0.806
				225	1.25
	37% Ni/MgO			150	2.34E-02
				170	7.49E-02
				195	0.141
				220	0.239
				240	0.375
				270	0.787

Results and Discussion

CO_2 hydrogenation activity of Co/CeO_2

Table S2. *Catalyst loading as determined by ICP-OES and metal particle size as determined by TEM and CO-chemisorption.*

Catalyst	Co metal loading (%)	Particle size (nm)	
		TEM	CO chemisorption
Co/SiO_2	8.8	6 ± 1	12[a]
Co/CeO_2	9.4	8 ± 3	7

[a] The larger particle size as determined by CO chemisorption compared to TEM for Co/SiO_2 is believed to be caused by an incomplete reduction of Co as confirmed by hydrogen temperature-programmed reduction (Figure S16). Consequently, the less CO is adsorbed leading to a perceived larger particle size.[34] For Co/CeO_2, the size determined by the two techniques are in good agreement.

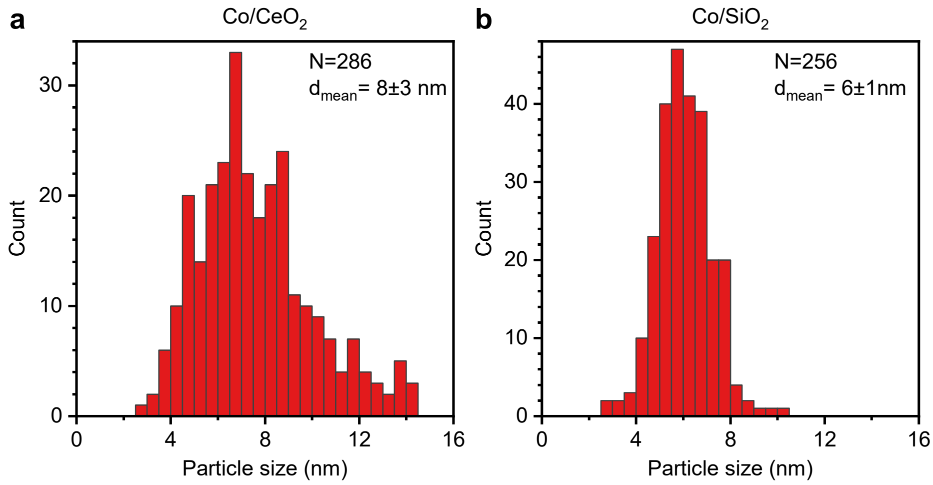

Figure S3. *Particle size distribution as determined by TEM for a) Co/CeO_2 and b) Co/SiO_2. Over 250 Co nanoparticles were measured for both Co/CeO_2 and Co/SiO_2, of which the mean size was similar for both catalysts.*

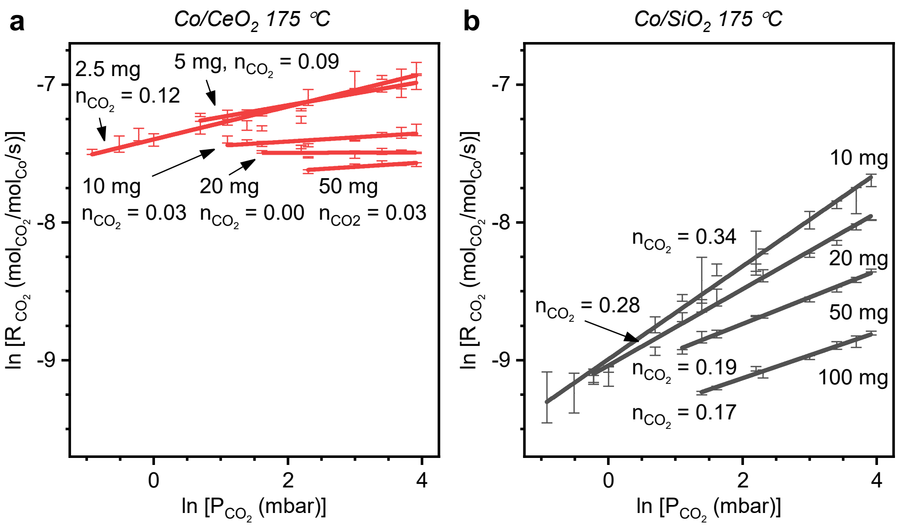

***Figure S4**. Reaction orders with respect to CO_2 ($n_{CO2} \pm 0.01$) for Co/CeO_2 (a) and Co/SiO_2 (b) at 175 °C with CO_2 partial pressures of 0.4–5 mbar. Reaction rates are expressed as specific reaction rates ($mol_{CO2}/mol_{Co}/s$). Error bars indicate the standard deviation between GC measurements (CO_2 conversions <10%, except 2.5 mg Co/CeO_2 at 400 ppm). Conditions: 2.5–100 mg catalyst, 0.4–5 mbar CO_2 and 200 mbar H_2 in He, total flow 200 ml/min.*

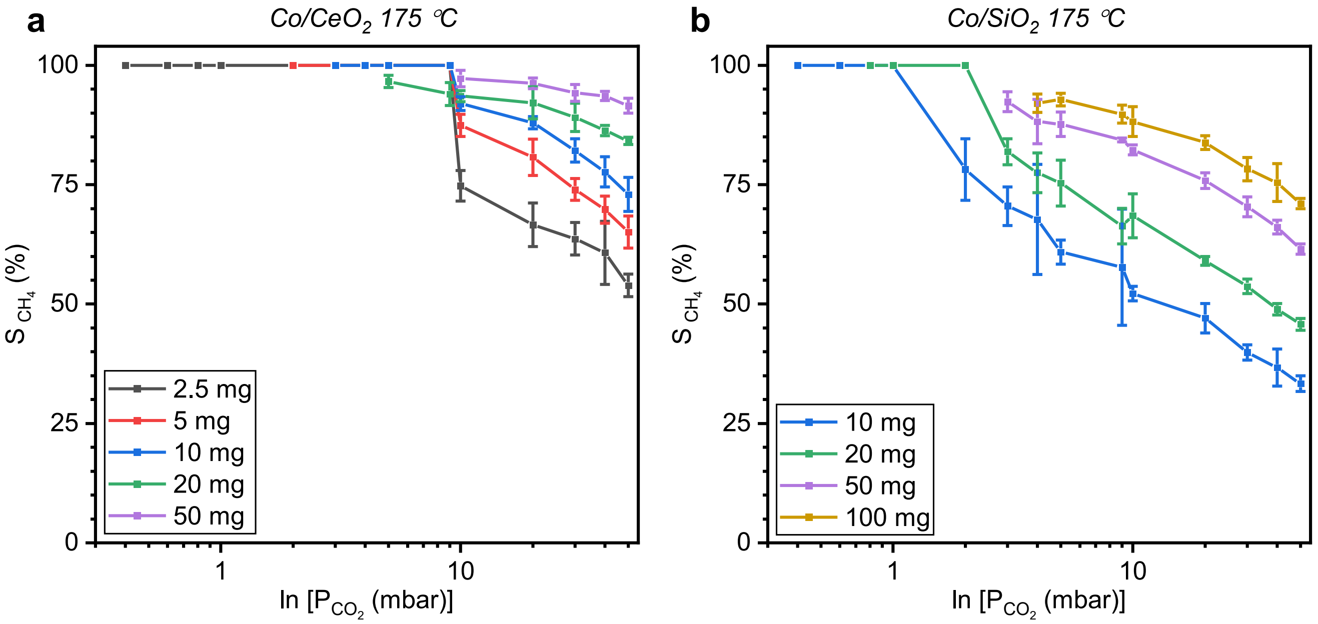

***Figure S5.** Selectivity towards CH_4 for a) Co/CeO_2 and b) Co/SiO_2 at 175 °C with CO_2 partial pressures varying from 0.4 to 5 mbar. A selectivity of 100% indicates that no CO was detected. The CH_4 selectivity was significantly lower for the same catalyst loadings of Co/SiO_2, which demonstrates a higher selectivity towards CO, for all partial pressures of CO_2 measured. The error bars indicate the standard deviation between GC measurements (all at CO_2 conversions below 10%, except for 2.5 mg Co/CeO_2 at 400 ppm due to high catalyst activity and low partial pressure of CO_2 even with low catalyst amount). For all measurements, He was used to obtain a total flow of 50 ml/min containing 200 mbar H_2 besides CO_2. Catalyst loadings of 2.5, 5, 10, 20, and 50 mg for Co/CeO_2 and 10, 20, 50, and 100 mg for Co/SiO_2 were used.*

Operando FTIR Spectroscopy at Low and High Partial Pressures of CO_2

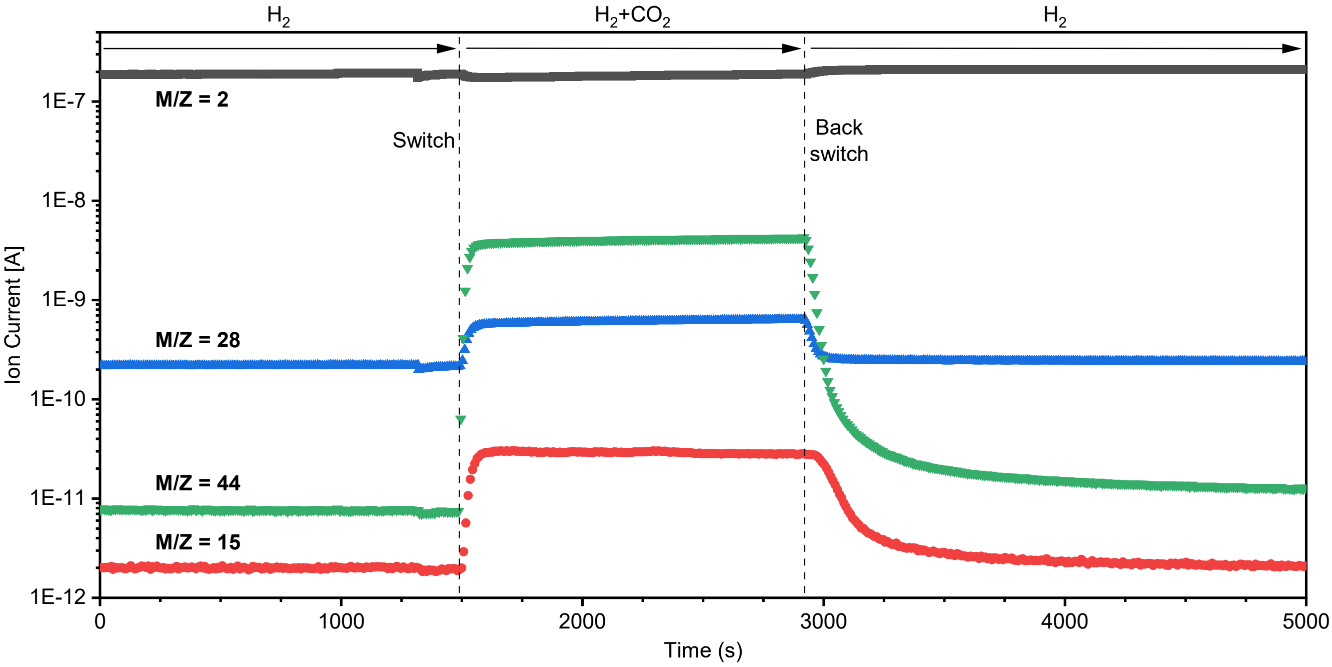

Figure S6. *Mass spectrometry data of the FTIR experiment at 25 mbar CO_2 for Co/CeO_2. Similar catalytic activity was observed before and after the switch from H_2 to CO_2+H_2. After the back switch, the CO production (followed by m/z = 28) decreased concurrently with the CO_2 (m/z = 44) whereas the CH_4 production (m/z = 15) only decreased after ~50 s. Conditions: 25 mbar CO_2 / 200 mbar H_2 balanced with He, total flow 200 ml/min.*

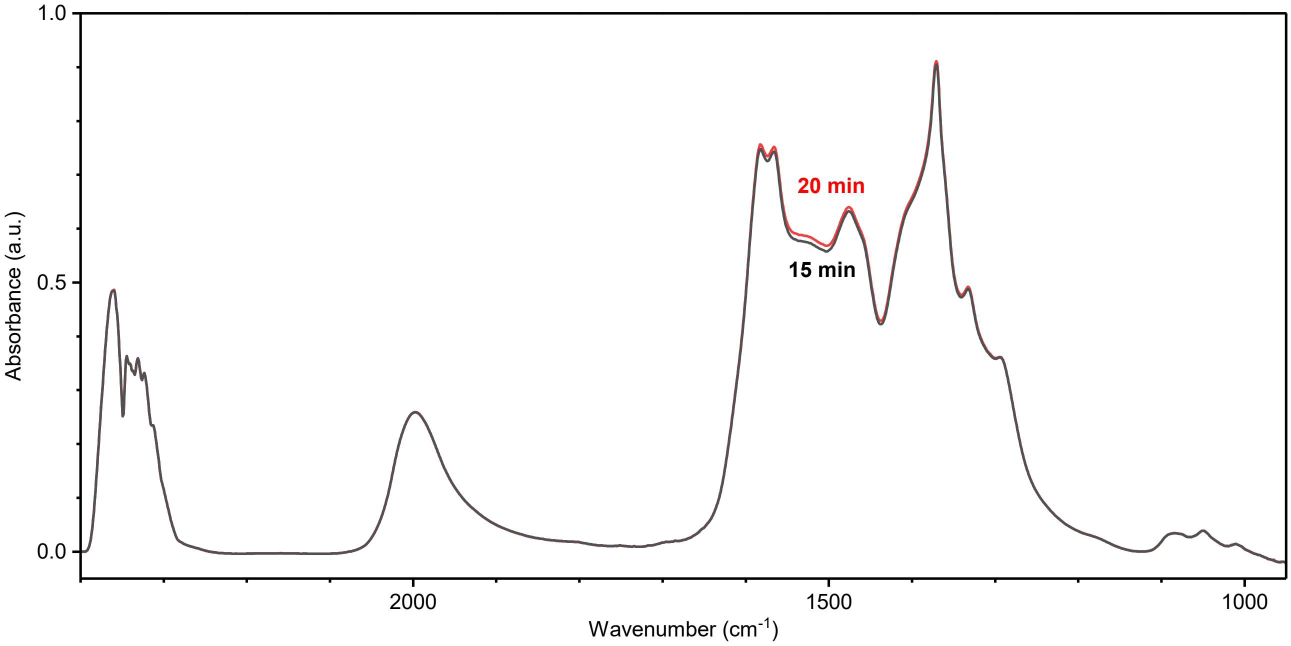

Figure S7. *FTIR spectra for Co/CeO_2 of measurement after 15 and 20 minutes after introduction of 25 mbar CO_2. No significant changes were observed for the species of interest. The small deviations 1400-1600 cm^{-1} are attributed to spectator carbonate species with a timescale that is irrelevantly long for the FTIR experiments. Conditions: 25 mbar CO_2 / 200 mbar H_2 balanced with He, total flow 200 ml/min.*

Note S1. Pressure-Dependency of Surface Coverages

Increasing the CO_2 partial pressure led to a significant increase in the carbonyls, formates, and carbonates on Co/CeO_2 (Figure 3a). For example, the carbonyl bands increased by more than 400%. By deconvolution of the absorption bands, it was determined that both linear and multibonded carbonyls (bridged- and hollow-CO) contributed to this significant increase. The ratio of linear- to multibonded-CO approximately remained constant upon increasing the partial pressure of CO_2. In contrast, the ratio of carbonyls to formates changed as the intensity of the formate bands increased only ~200% instead of >400%. The observed difference in the abundance of the species indicates the importance of studying the catalysts at the reaction conditions of interest (in this case low CO_2 pressure).

For Co/SiO_2 the ratio between the carbonyls and formates as well as between the individual carbonyl species changed (Figure 3b). The number of formates present under steady-state with 25 mbar CO_2 in the feed increased by 200% compared to 0.6 mbar CO_2. The carbonyls increased substantially less on Co/SiO_2 than on Co/CeO_2 (a ~75% and >400% increase of the quantity at 0.6 mbar, respectively), primarily due to the linear carbonyls. While for Co/CeO_2 the bridged/hollow-CO increased substantially, their increase was minimal on Co/SiO_2.

The normalized carbonyl intensity (insets Figure 3) demonstrates another important benefit of studying catalysts at low partial pressures. At 0.6 mbar CO_2, an additional top-CO peak is observed as a clear blue-shifted shoulder. Moreover, the bridged-CO resulted in a shoulder on the low wavenumber side. At 25 mbar CO_2, on the other hand, it was not possible to observe or deconvolute the additional linear species. These carbonyl species are probably still present in a small quantity, hidden by the large top-CO peak at 25 mbar CO_2. Furthermore, the lateral blue-shift of this large peak most probably resulted in an overlap with the additional carbonyl peak.

Probing Oxidation, Carburization and Hydridization of Co/CeO$_2$

Note S2. Pressure- and Time-Dependent CO Adsorption Experiments

To investigate the carbonyls, including the characteristics and origin of the additional peak, CO adsorption experiments were performed on Co/SiO$_2$ and Co/CeO$_2$ and monitored by FTIR spectroscopy. The self-supporting pellets were first reduced in situ at 500 °C for 4h in 200 mbar H_2 in He. Subsequently, the cell was evacuated at a high vacuum to remove adsorbed species and cooled down to 50 °C in vacuum. At 50 °C, FTIR spectra were acquired at partial pressures of CO between 0 and 10 mbar.

Dosing CO to Co/CeO$_2$ resulted in the increase of carbonyls with a rising partial pressure of CO (Figure S8). An increase in intensity and a significant shift of the maximum absorbance for linear carbonyls on Co/CeO$_2$ was observed up to 0.03 mbar CO. Further increase of P_{CO} resulted in only minor intensity changes for the linear carbonyls while the peak shifted even further to 2047 cm^{-1}. The absorbance of two other wavenumber regions increased in addition to linear-CO at higher partial pressures of CO. Firstly, the intensity of bridged carbonyls around 1900 cm^{-1} increased. Secondly, peaks in the regions of 1650-1200 cm^{-1} and 1100-950 cm^{-1} appeared. These bands are attributed to several carbonous species as carbonates, hydro carbonates, and formates.[20,35]

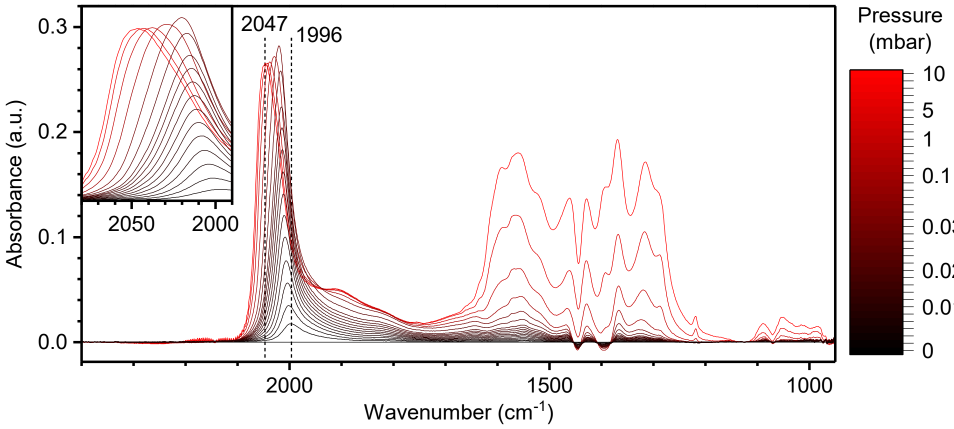

Figure S8. *CO adsorption on Co/CeO$_2$ at 50 °C after a 4h reduction at 500 °C. The spectra were obtained by subtracting the spectrum of the reduced sample and normalization by pellet weight.*

The blue-shift of the linear carbonyl peak indicates a weakening of the CO binding. A change in the cobalt surface can explain the weaker bonding upon adsorbing the CO. This change might be caused by the presence of adsorbed species or by a partial re-oxidation of the surface. A shift of the carbonyl band can be explained by repulsive lateral dipole-dipole interactions with other adsorbed carbonyls upon an increasing CO surface coverage.[36] For example, a blue-shift of 20-25 cm^{-1} was attributed to this phenomenon in the literature upon increasing the CO surface coverage from approximately 0 to 1 on Co/SiO$_2$.[37] In this work, the shift from 1996 to 2021 cm^{-1} upon increasing the CO partial pressure up to 0.03 mbar can be caused by this phenomenon.

Adsorption on sites adjacent to C_{ads} or O_{ads} atoms on the cobalt surface is also known to lead to weaker CO adsorption.[36,38,39] Possible processes leading to the carburization of the surface can be I) the disproportionation of two *CO molecules into C_{ads} and CO_2 (Boudouard reaction); II) the dissociation of *CO into C_{ads} and O_{ads}, which both remain on the cobalt surface, and III) the dissociation of *CO in which the C_{ads} remains on the Co surface while the oxygen either oxidizes the metal or the CeO$_2$ support via

oxygen spillover. For instance, carburization was reported to cause the shift of the linear carbonyl band on Co/SiO_2 from 2034 to 2063 cm^{-1}.[36] Phenomenon I has been reported in the literature on multiple supports, e.g. SiO_2, Al_2O_3, and CeO_2-SiO_2.[40] Lastly, co-adsorption of CO on a Co atom with a hydrogen atom (HCoCO, called a hydrocarbonyl) was reported to result in a band between 2070-2030 cm^{-1}.[40,41]

Besides the presence of adsorbed surface species, a re-oxidation of the cobalt surface can result in the observed blue-shift. As indicated, process III could enable a partial re-oxidation in the presence of CO. CO_2 can also provide these oxygen atoms upon adsorption under reaction conditions. Re-oxidation can result in an increase in wavenumber as shown by CO adsorption on Co^{2+}, Co^{+}, and Co.[37,38,41] Moreover, CO adsorption on cobalt with a partial positive charge ($Co^{\delta+}$) has been proposed as a band around 2060 - 2050 cm^{-1}.[41,42]

The Boudouard reaction (CO disproportionation to CO_2) is likely the main pathway for the CO_2 formation on the fully reduced samples. As the sample was reduced in situ at 500 °C, it can be assumed that CO cannot react with oxygen from the CeO_2 support via the reverse oxygen spillover mechanism.[43–45] The CO_2 formed on the cobalt surface can either migrate to the support or desorb followed by re-adsorption on CeO_2, giving rise to the carbonous species observed at the higher CO pressures.

Besides CO_2, C_{ads} remains on the cobalt surface in this disproportionation reaction. Synchronous 2D correlation analysis demonstrates a perfectly synchronous appearance of the bands in the carbonate region (1650-1200 cm^{-1}) with the shift of carbonyl species from 2021 to 2047 cm^{-1} (Figure S9). Therefore, the shift may be caused by the adsorption of CO next to an adsorbed carbon atom formed by the Boudouard reaction (C_{ads}-CO).

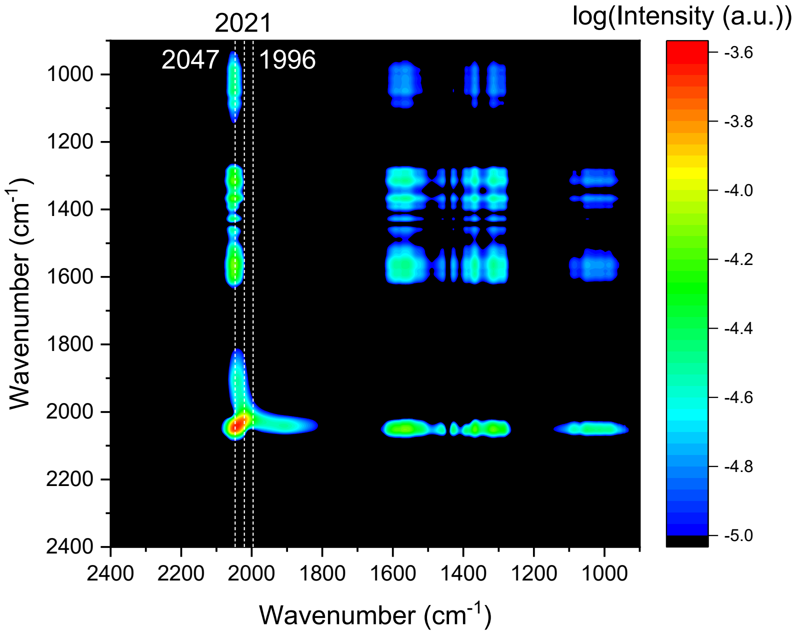

Figure S9. *Synchronous 2D correlation for the dosed adsorption of CO on Co/CeO_2 (Figure S8). Lines are indicated for the maxima of the linear CO peak at low P_{CO} (1996 cm^{-1}), at the moment of maximum intensity (2021 cm^{-1}), and at 10 mbar (2047 cm^{-1}).*

Similar to Co/CeO_2, a shift of the linear carbonyls is observed for CO adsorption on Co/SiO_2 (Figure S10). Although the position shift was similar (approximately 50 cm^{-1}), the shape of the linear CO peak broadens

significantly for SiO_2, where it remained rather sharp for CeO_2. By deconvolution, a clear increase of an additional peak over an increase of partial pressure can be observed (Figure S11).

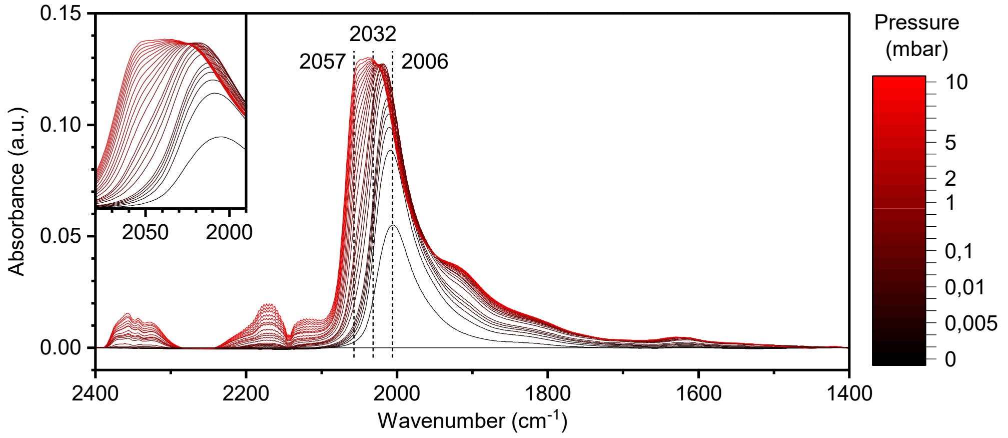

Figure S10. *CO adsorption on Co/SiO₂ at 50 °C after 4h reduction at 500 °C. The spectra were corrected for peaks present before adsorption by subtracting the scan taken before adsorption and normalized by pellet weight.*

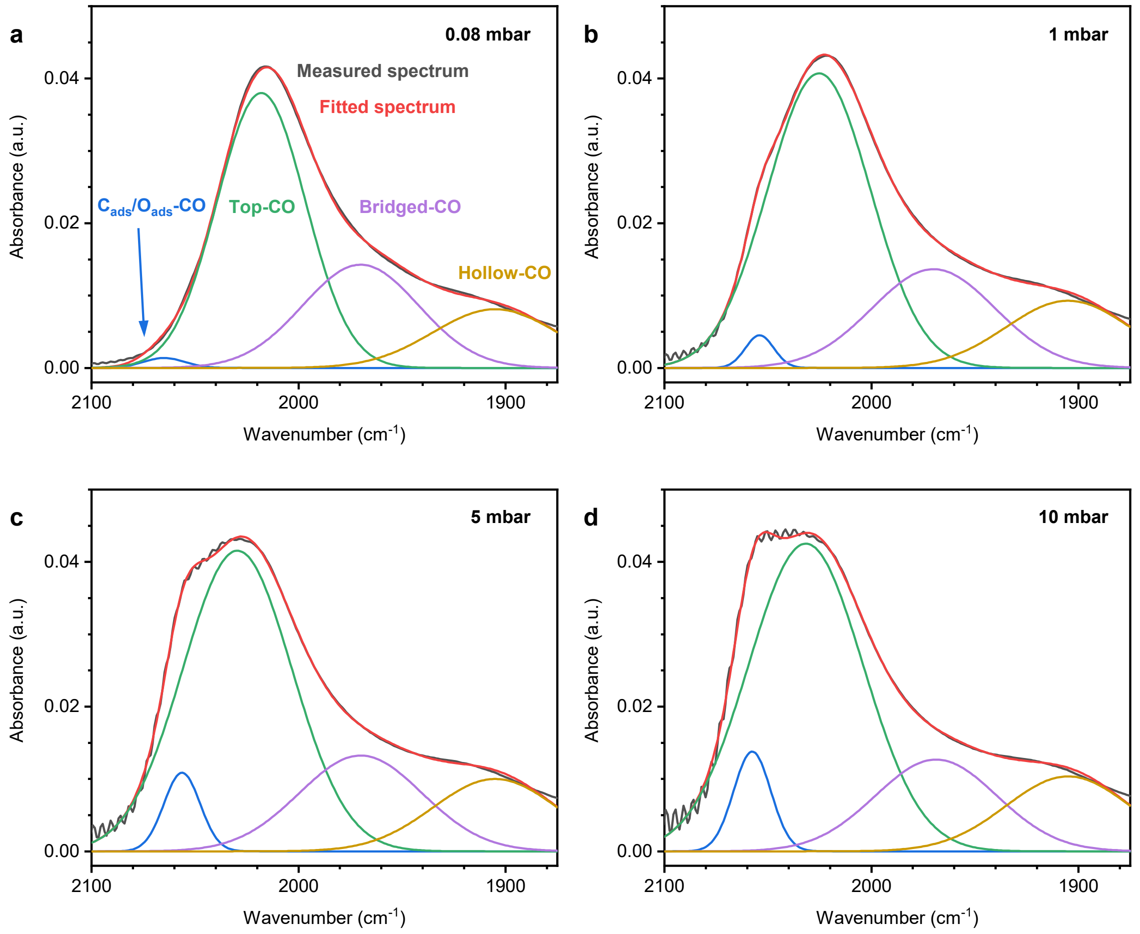

Figure S11. *Deconvoluted spectra for the dosed adsorption of CO on Co/SiO₂ (Figure S10). Four Gaussian-shaped contributions were taken into account; 1) linear carbonyls (peak maximum between 2050-1990, maximum std dev. 30); 2) extra carbonyl peak (peak maximum between 2065-2045, maximum std dev. 10); 3) bridged carbonyls (peak maximum between 1970-1650, maximum std dev. 30) and; 4) multi-bonded carbonyls (peak maximum between 1945-1905, maximum std dev. 30).*

Another difference from Co/CeO_2 is the absence of carbonous species in the carbonate region on Co/SiO_2, except for a small formate peak around 1625 cm^{-1}.[46] Instead, the SiO_2 supported catalyst showed peaks between 2300-2400 cm^{-1} representative of gaseous CO_2.[40] The presence of this gaseous CO_2 confirms the disproportionation of CO on Co/SiO_2 and thus the carburization of the surface. As the SiO_2 support is an irreducible oxide, the sample itself cannot supply the oxygen for CO_2 formation. The simultaneous appearance of the additional peak and CO_2 was again confirmed by 2Dcor analysis (Figure S12). Therefore, it is hypothesized that also for Co/SiO_2 the Boudouard reaction contributes to the peak appearing at 2057 cm^{-1}.

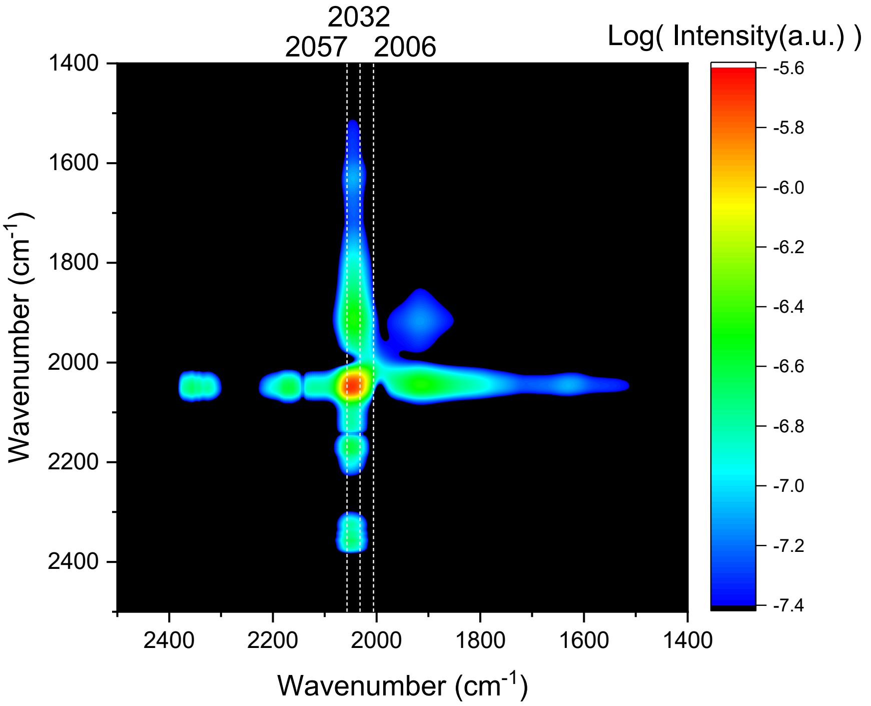

Figure S12. *Synchronous 2D correlation for the dosed adsorption of CO on Co/SiO_2 (Figure S10). Lines are indicated for the maxima of the linear CO peak at low P_{CO} (2006 cm^{-1}) and the positions of the linear carbonyls (2032 cm^{-1}) and the second peak (2057 cm^{-1}) in the fitted spectra at 10 mbar.*

Alternative explanations for the blue-shifted extra peak in these CO adsorption experiments could not be ruled out at this point. The weak adsorption of CO on unreduced cobalt present before exposure to CO could explain an increase of the observed extra peak upon increasing P_{CO}. Alternatively, co-adsorption with a hydrogen atom (present from the reduction step) blue-shifts the absorption band of *CO. Time-dependent experiments can be used to exclude these possibilities. Indeed, these phenomena would not cause an increase of the extra peak intensity over time, in contrast to the aforementioned phenomena (C_{ads}/O_{ads}-CO). In these experiments, a CO partial pressure of 0.1 and 0.5 mbar was dosed at once to the cell. The peaks were subsequently observed for 70 minutes to investigate the presence of a time dependency for both Co/CeO_2 and Co/SiO_2.

For both catalysts at 0.1 mbar as well as 0.5 mbar (Figure S13), the phenomenon proved to be time-dependent. For Co/CeO_2 at both pressures, the peak shifts to higher wavenumbers over time. The amount of the shift was approximately 15 wavenumbers for both catalysts. However, the initial position was higher for 0.5 mbar than for 0.1 mbar; 2027 and 2020 cm^{-1}, respectively. As for the initial measurements the exposure to CO was very short, the difference in the start position of the carbonyls can be ascribed to

the lateral interactions as discussed before. For Co/SiO_2, the peak that was previously observed (~2057 cm^{-1}) appeared to a minor extend at 0.1 mbar, while for 0.5 mbar a more prominent contribution was observed (~2050-2055 cm^{-1}). The start position of the top-CO was 5 cm^{-1} higher at 0.5 mbar versus 0.1 mbar as well, again ascribed to increased lateral interactions.

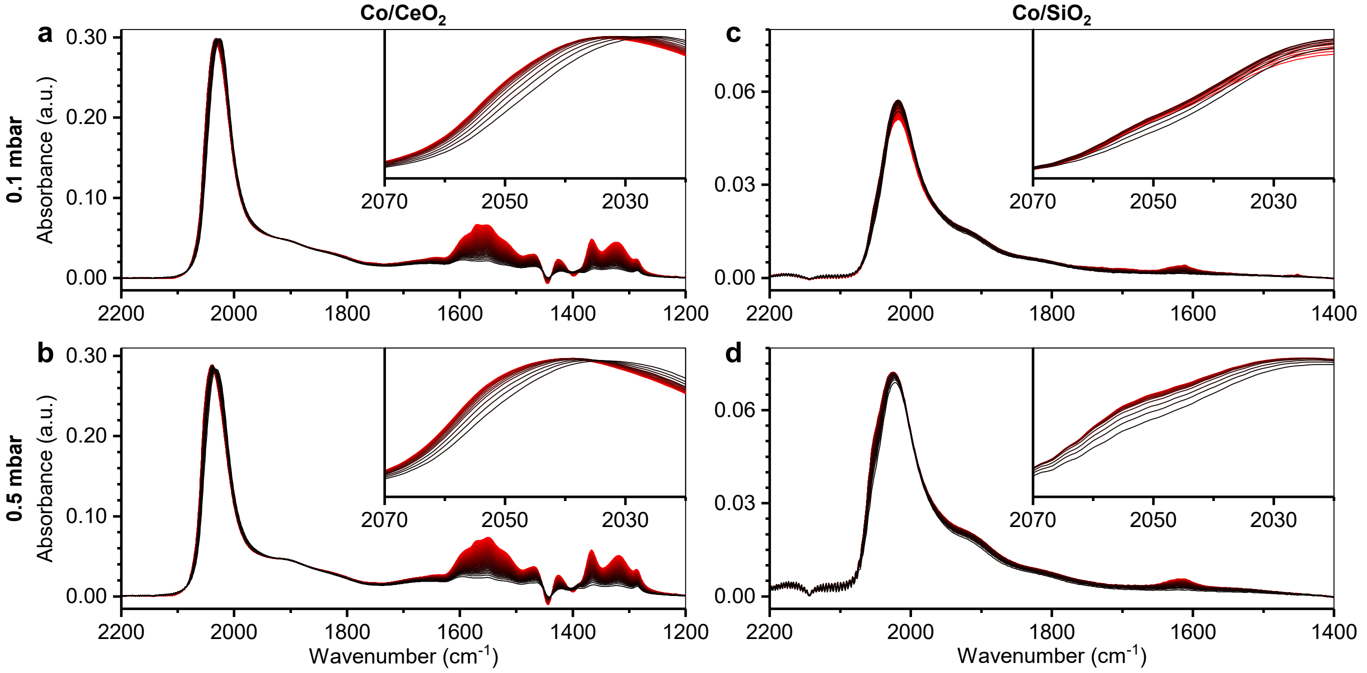

Figure S13. *CO adsorption experiment on Co/CeO_2 at a) 0.1 mbar and b) 0.5 mbar; and on Co/SiO_2 at c) 0.1 mbar and d) 0.5 mbar of CO. All spectra were corrected by subtraction of a scan taken before adsorption. The absorbance for each spectrum was normalized by pellet weight. Conditions: 50 °C, 4h reduction at 500 °C.*

In summary, the combination of both pressure- and time-dependent experiments leads to the conclusion that the appearance of the extra peak is dependent on both the partial pressure of CO and time. The observed time dependency rules out the adsorption on unreduced cobalt sites or next to adsorbed atoms present prior to CO exposure as the cause for the additional peak. Indeed, the weak adsorption of CO on these or other sites would depend on the partial pressure but not on time. Consequently, the observed linear-CO peak on higher wavenumbers than top-CO is linked to the dissociation/disproportionation of CO. The appearance of the blue-shifted carbonyl band is then caused by either the CO adsorption adjacent to C_{ads}/O_{ads} and/or a partial surface oxidation by O_{ads}.

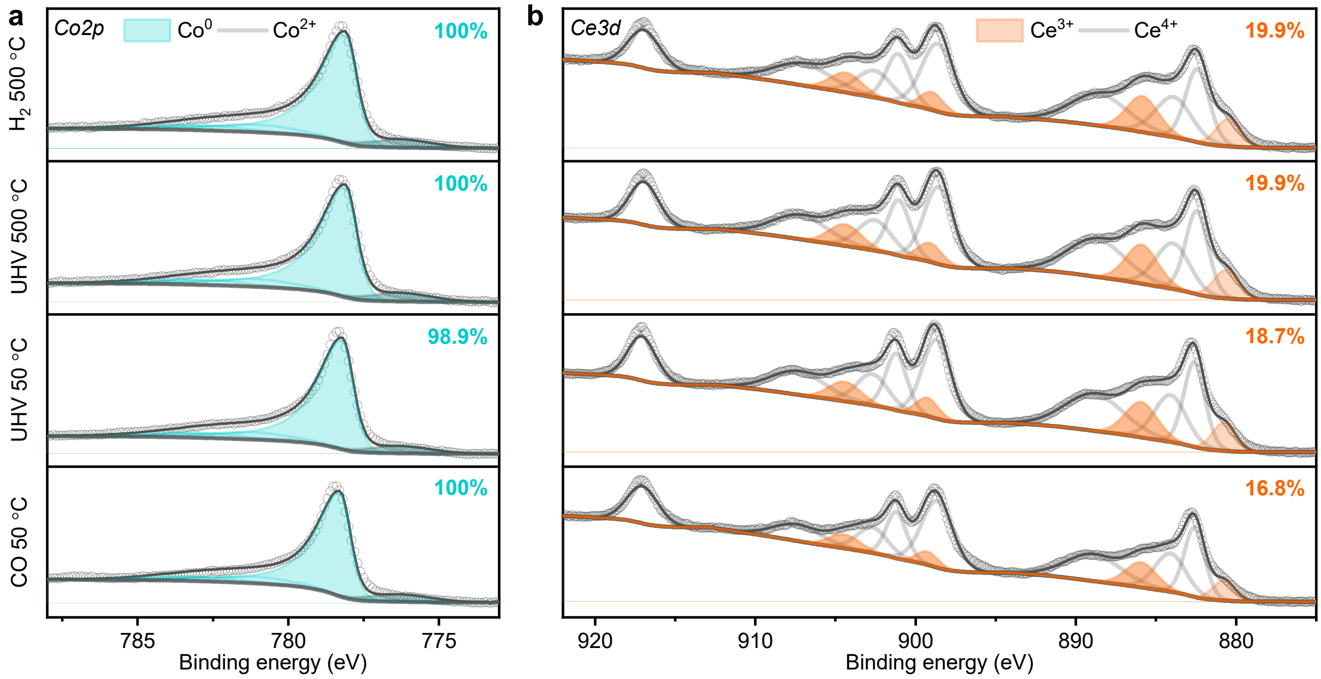

Figure S14. *CO adsorption operando lab-based NAP-XPS study on Co/CeO_2 for a) the Co 2p and b) the Ce 3d region. The Co LMM contributions are indicated by the grey area. The top row was acquired after reduction at 500 °C in H_2. Cooling down from 500 °C to 50 °C was done under UHV. The catalyst was subsequently exposed to different temperatures and UHV or CO (upon exposure to CO the Co 2p line shifted by 0.1 eV). Upon exposing the sample to CO, no significant oxidation of cobalt was observed whereas for ceria a decrease of Ce^{3+} from 18.7% to 16.8% was observed, serving as an indication for of the occurrence of CO dissociation in which O_{ads} oxidizes the CeO_2 support via oxygen spillover. The presence of cerium hydrides did not result in an oxidation of Co upon exposure to CO, in line with these results (Figure S15).*

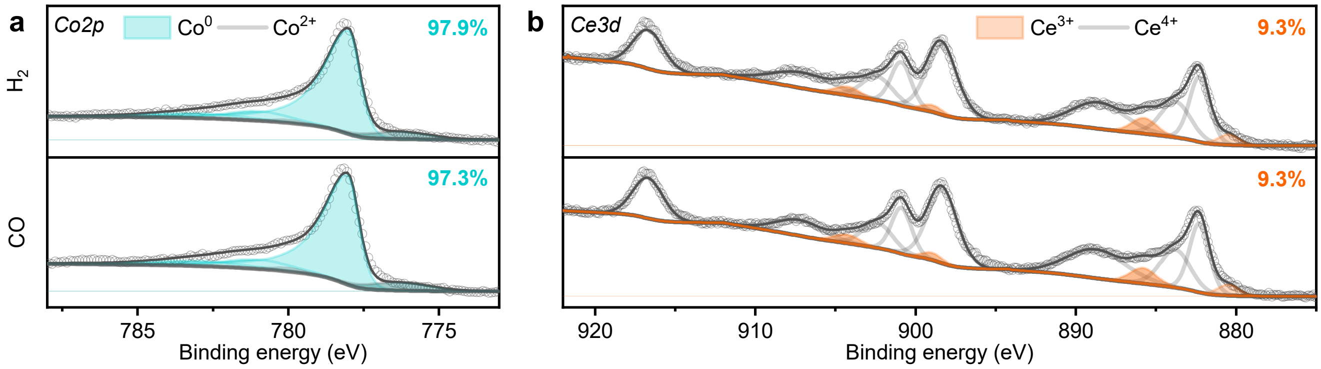

Figure S15 *CO dosing at 50 °C after reduction at 500 °C and cooling down in hydrogen. The top row was acquired after reduction at 500 °C in H_2 and cooling down from 500 °C to 50 °C under the same hydrogen atmosphere. As demonstrated by the bottom spectra, the oxidation state of Co (**a**) and Ce (**b**) did not change significantly after switching the feed to the NAP-XPS cell from H_2 to CO.*

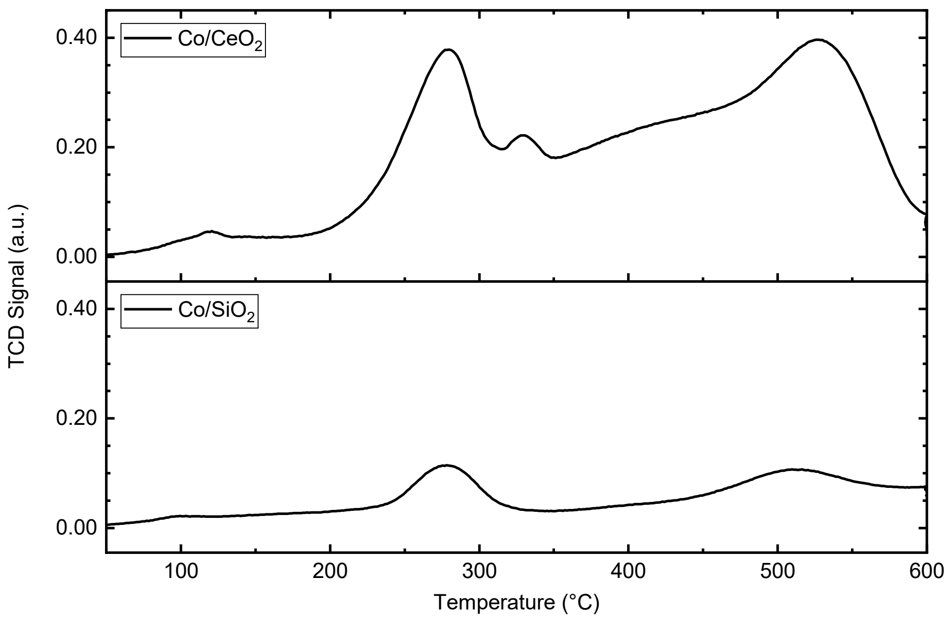

Figure S16. *Hydrogen temperature-programmed reduction (H_2-TPR) of Co/CeO_2 and Co/SiO_2. The TCD signal, proportional to the hydrogen consumption, is normalized for the used catalyst weight. These TPR profiles demonstrate the facile reduction of the CeO_2 support, as well as the typical step-wise reduction of Co_3O_4 for both catalysts.[47,48] The low temperature reduction (LTR) peak around 275 ˚C on both catalysts corresponds to the reduction of Co^{3+} to Co^{2+} and high temperature reduction (HTR) peak around 520 ˚C corresponds to the reduction of Co^{2+} to Co^0. For Co/SiO_2, the low hydrogen consumption indicates hard to reduce species and an incomplete reduction of all present cobalt. The Co that is not reduced might be present as cobalt silicates, known for their resistance against reduction.[49] The peak around 330 °C for Co/CeO_2 is ascribed to the reduction of the CeO_2 support.[50] The contributions connecting the 330 °C and 530 °C peaks represent the bulk reduction of ceria, potentially with contributions of the HTR of CoO. The broadness of this HTR peak can be caused by the presence of different cobalt species with different strengths of interactions with the support[9]. Additionally, the surface reduction of ceria could explain the minor feature seen between 100-150 °C.[51] Thus, (H_2-TPR) confirms the facile reduction of CeO_2 and thus the formation of oxygen vacancies. Besides the high basicity, the ability of the CeO_2 support to form oxygen vacancies is an important factor leading to the enhanced adsorption and activation of CO_2.[9,20,50,52]*

Note S3. Cerium Hydride Species

The apparent oxidation of Ce^{3+} to Ce^{4+} is ascribed to the formation of cerium hydride species ($Ce^{4+}H^{-}$) upon cooling down in hydrogen[53,54]. In recent years, the interaction of hydrogen with CeO_2 has been extensively studied given its catalytic activity in partial hydrogenations of alkynes[55–57]. Cerium hydride species are formed by the splitting of hydrogen on ceria followed by electron transfer from Ce^{3+} to hydrogen (resulting in oxidation of Ce^{3+} to Ce^{4+}). Cerium hydride species can even migrate from the surface to the bulk[58]. Direct evidence for these surface and bulk hydrides was provided by Wu et al. using neutron scattering[59].

Hydride formation was predicted to be thermodynamically favorable below ~665 K and the rate of hydride formation was reported to increase with increasing oxygen vacancy concentrations and decreasing temperatures[60,61]. Consistent with these results, the apparent oxidation of Ce continued upon further cooling from 150 to 50 °C in H_2 (Figure S18). The temperature-induced migration of oxygen vacancies to the bulk, can be considered as an alternative cause for the decrease in the Ce^{3+} concentration.[58] However, the Ce^{3+} concentration remained high after cooling down to 50 °C in UHV (18.7% Ce^{3+}, Figure S14). This finding emphasizes the role of gaseous hydrogen in the formation of surface hydrides accompanied by the decrease of Ce^{3+} surface. Thus, the observed decrease in Ce^{3+} concentration does not imply a loss in oxygen vacancies but is related to the formation of cerium hydrides. In addition to existing reports on cerium hydride formation on bare[54] or doped CeO_2,[56] our NAP-XPS data indicate the possibility of hydride formation on CeO_2 with deposited cobalt nanoparticles.

We should note that, these cerium hydrides likely decompose upon exposure to CO_2 in the reaction mixture and therefore are not present at steady state reaction conditions.[61,62] The presence of cerium hydrides is not a prerequisite for efficient CO_2 dissociation on Co/CeO_2. Even in the absence of these species, *i.e.* after cooling the sample in vacuum, the CO_2 dissociation can occur as confirmed by the appearance of carbonyls, in particular C_{ads}-CO, in FTIR spectra (Figure S17).

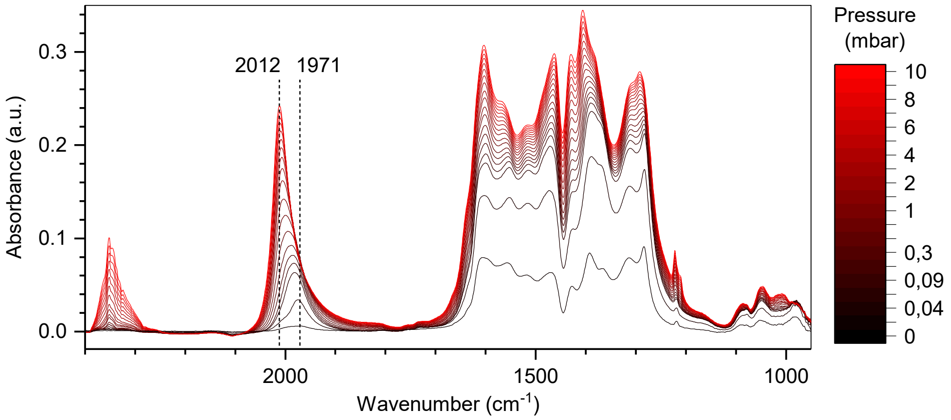

Figure S17 *CO_2 adsorption on Co/CeO_2 at 50 °C after 4h reduction at 500 °C and cooling down in vacuum. The spectra were corrected for peaks present before adsorption by subtracting the scan taken before adsorption and normalized by pellet weight. Upon dosing gaseous CO_2 to the FTIR cell (identified by the bands between 2300-2400 cm^{-1}), carbonyls were formed (1750-2100 cm^{-1}) including C_{ads}-CO (which can be identified as a separate peak already at low P_{CO2}) demonstrating CO_2 dissociation. Additionally, multiple other carbon species are formed upon adsorption of CO_2 on ceria (1650-1200 cm^{-1} and 1100-950 cm^{-1}).*

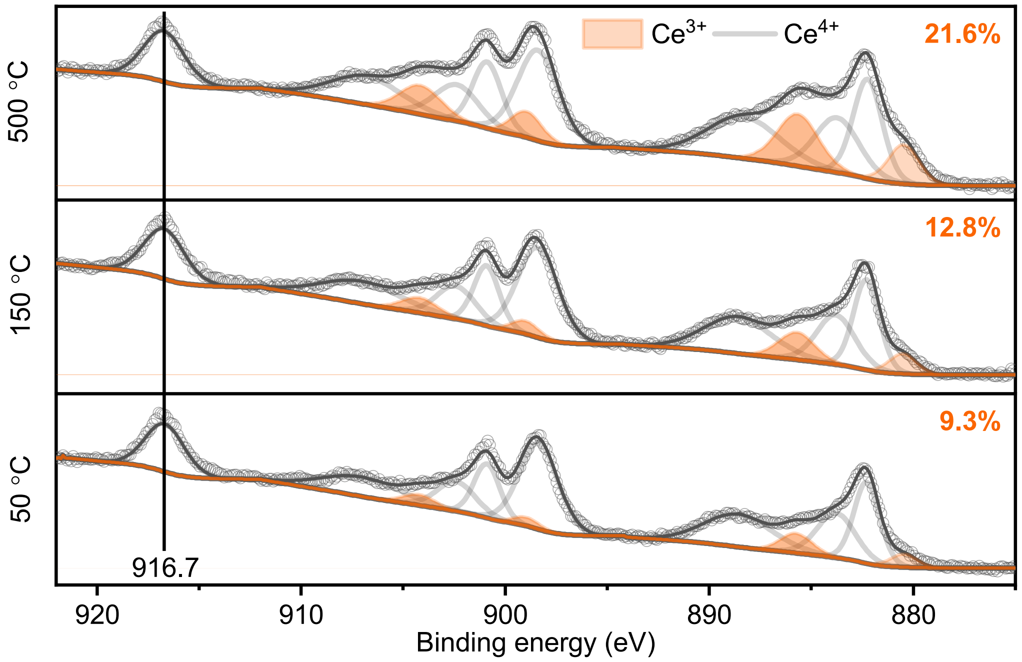

Figure S18 *Operando lab-based NAP-XPS study on Co/CeO_2 in hydrogen at different temperatures for the Ce 3d region. The top row was acquired after reduction at 500 °C in H_2. Spectra were subsequently acquired after cooling down to 150 °C and 50 °C in H_2. A significant decrease in Ce^{3+} over temperature is observed that is ascribed to the formation of cerium hydride species ($Ce^{4+}H^-$).*

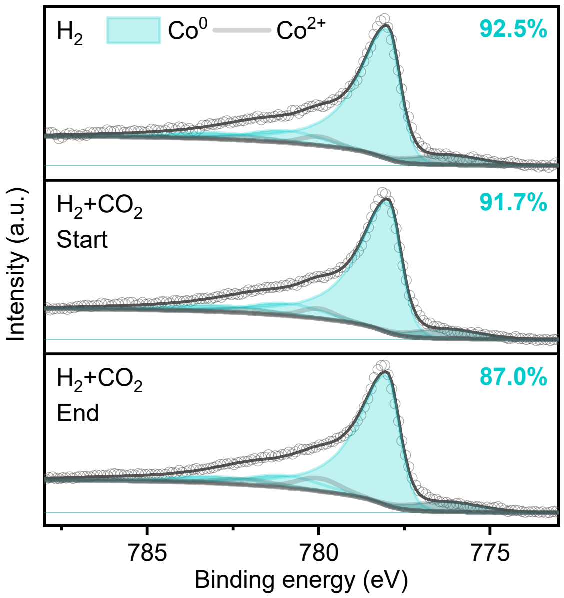

Figure S19 *NAP-XPS on Co/CeO_2 at 150 °C. The gas composition is changed from H_2 (the top graph) to H_2+CO_2. After the switch, the oxidation gradually takes place over time (the middle graph) towards the steady state oxidation degree (the bottom graph).*

Steady-State Operando FTIR Spectroscopy

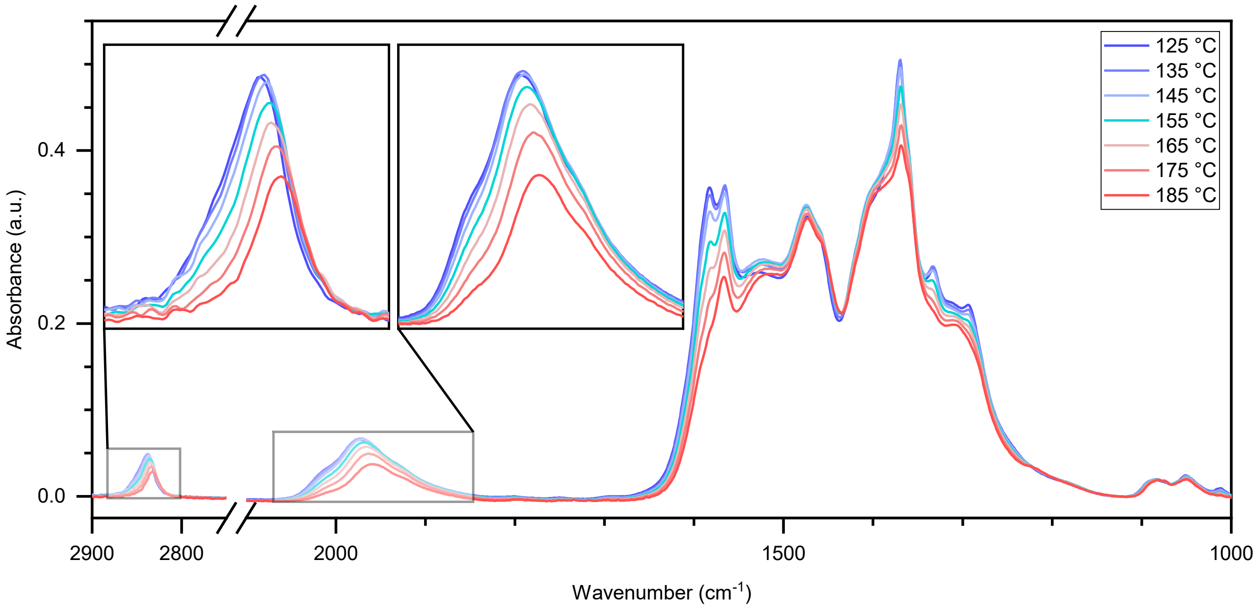

Figure S20. *Steady-state FTIR scans at reaction conditions on Co/CeO_2. Upon changing the temperature from 125 ˚C to 185 ˚C, the formates decrease as demonstrated by the formate peaks in the formate C-H stretch region (left inset, 2800-2900 cm^{-1}) and formate C-O stretch regions (1550-1600 cm^{-1} for C-O stretch I and 1350-1370 cm^{-1} for C-O stretch II).[35] The carbonyls were also found to decrease as demonstrated by peaks in the carbonyl region (right inset, 1800-2100 cm^{-1}). Conditions: 0.6 mbar CO_2 / 200 mbar H_2/ balanced with He, total flow 200 ml/min, 125-185 °C.*

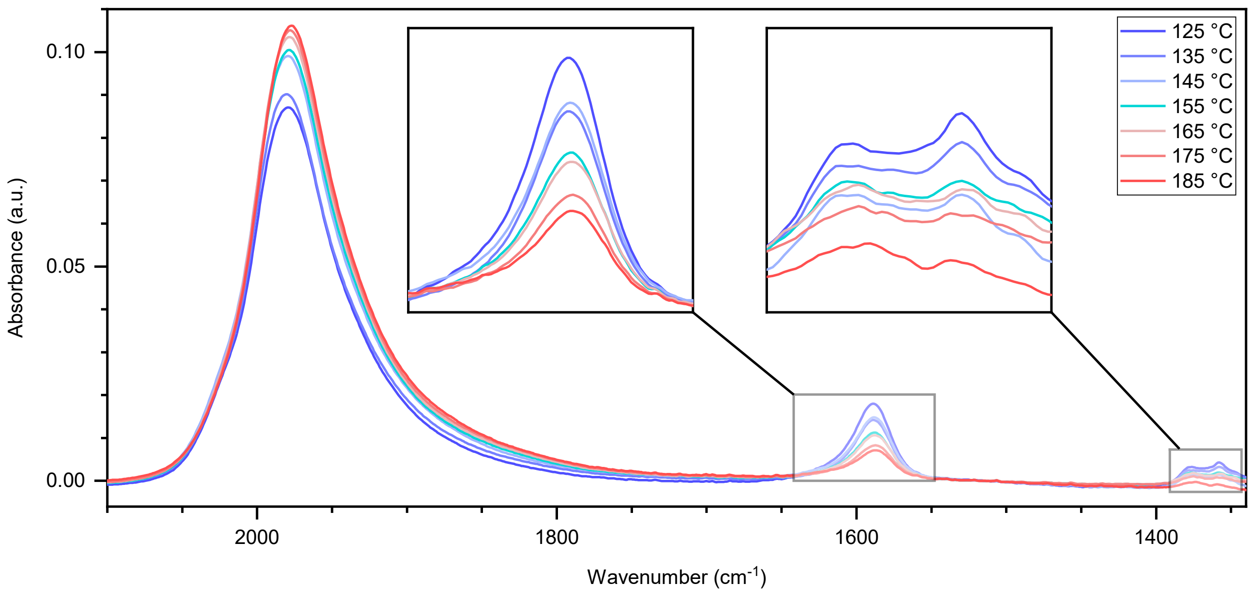

Figure S21. *Steady-state FTIR scans at reaction conditions on Co/SiO_2. Upon changing the temperature from 125 ˚C to 185 ˚C, the formates decrease as demonstrated by the formate peaks in the formate C-O stretch regions (1550-1600 cm^{-1}, left inset, for C-O stretch I and peak at 1377 cm^{-1}, right insert, for C-O stretch II) and the O-C-H bending region (peak at 1358 cm^{-1}, right inset).[35] The carbonyls were found to increase as demonstrated by peaks in the carbonyl region (1700-2100 cm^{-1}). Conditions: 0.6 mbar CO_2 / 200 mbar H_2/ balanced with He, total flow 200 ml/min, 125-185 °C.*

Note S4. 2D Correlation Assisted Determination of Formate Species on CeO_2

The presence of two types of formates was identified in the steady-state experiments on Co/CeO_2. The sets of vibrations for these formates were found around 2855/2838 cm^{-1} for the C-H stretching vibration, 1584/1565 cm^{-1} for one C-O stretching vibration, and 1369/1359 cm^{-1} for another C-O stretching mode. By the use of a H_2/D_2 SSITKA-FTIR experiment, these vibrations were unequivocally assigned to formates. Figure S22 displays the spectra in a reaction mixture containing hydrogen and in deuterium. The C-H stretching vibrations shifted by approximately 700 cm^{-1} to 2160/2145 cm^{-1}, characteristic of this formate vibration.[35] Moreover, both C-O stretching vibration sets shifted to lower wavenumbers as expected. Two additional features became visible around 1000 cm^{-1}. This set is ascribed to the formate O-C-H bending mode. Under a reaction flow containing hydrogen, these vibrations were likely not observed due to the large overlap with the carbonate peaks.[35]

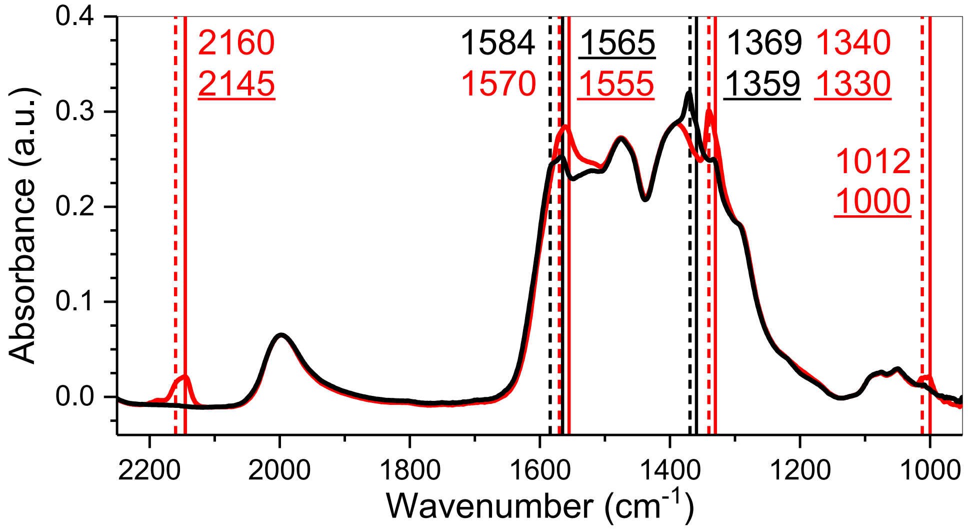

Figure S22. *Spectrum under reaction atmosphere containing H_2 (black) and D_2 (red) at 175 °C on Co/CeO_2. The wavenumber position for each set of formate vibrations is indicated by lines. For clarity, the low wavenumber contributions are indicated by a solid line (positions underlined) while the high wavenumber contribution is dashed (not underlined).*

Both the auto- and cross-peaks demonstrated the position and correlation of the formates in the 2D correlation analysis on this SSITKA. Moreover, it showed that the H/D exchange rates were relatively similar for both formates, indicating a good hydrogen supply for both formate types (Figure S23). Additionally, the 2D correlation demonstrated the presence of hydrogen carbonates. Due to strong overlap with an intense and broad carbonate peak around 1573 cm^{-1}, these contributions initially were hard to identify. However, the synchronous 2Dcor spectrum clearly shows the presence of two different types of bicarbonates.[35] The C-O stretch vibrations of these bicarbonates were present around 1616 and 1592 cm^{-1}.

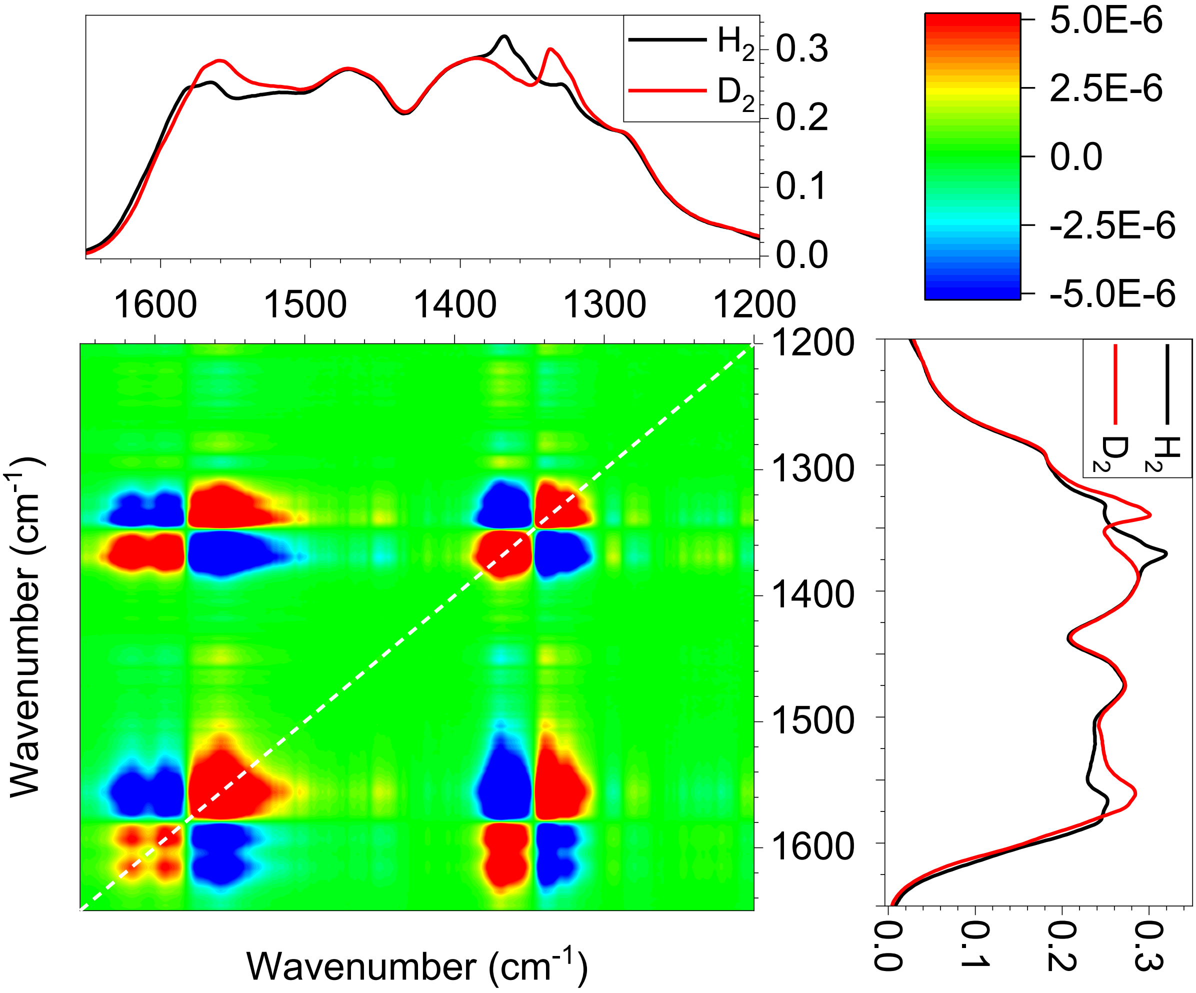

Figure S23. *Synchronous 2D correlation of the H/D exchange at 25 mbar CO_2 and 175 °C on Co/CeO_2. Steady-state spectra under reaction atmosphere containing H_2 (black) and D_2 (red) are given on the top and right.*

Lastly, 2D correlation analysis was also applied to the results of the transient kinetic step response switches. These analyses supported the conclusion that the formates are different in nature as proven by their different reaction rates. Examples of a synchronous and asynchronous 2Dcor map are provided in Figure S24 and Figure S25, respectively. The synchronous map shows the correlation between the gaseous CO_2 signal and the carbonate/formate region. As the gaseous CO_2 disappears fast upon the switch from a CO_2 / H_2 / He to a H_2 / He flow, peaks that correlate strongly are also fast. The formates at 1584 and 1565 cm^{-1} resulted in sharp features as expected for formates.[35] The features around 1369 and 1359 cm^{-1} also resulted in a sharp and intense peak but overlapped with each other. Already from the stronger correlation with CO_2, it can be deduced that blue-shifted formate is faster and thus likely the active species. The correlation of the carbonate/formate region was analyzed using asynchronous 2Dcor. Indeed, the formates with positions 1584 and 1369 cm^{-1} preceded the disappearance of the formate with positions 1565 and 1359 cm^{-1}, supporting the blue-shifted formate to be the dominantly active species. Combining all data, the peaks located at 2855, 1584, and 1369 cm^{-1} can be linked to one formate species (formate-I) and peaks located at 2838, 1565, and 1359 cm^{-1} to another formate (formate-II).

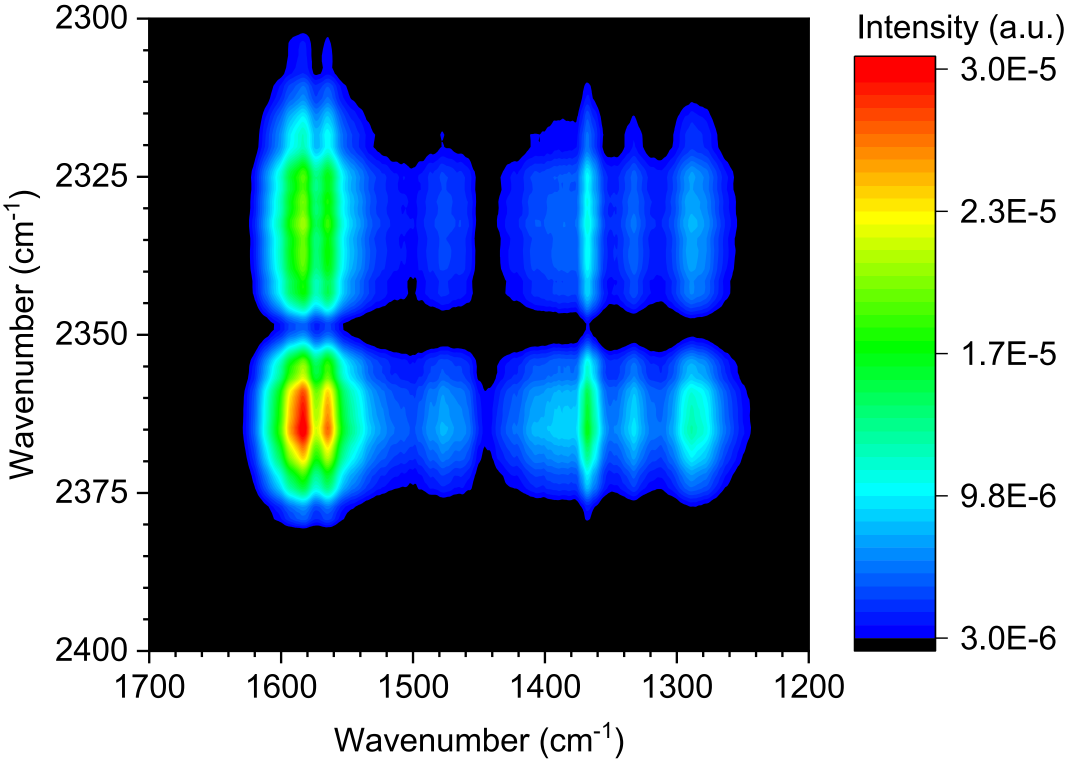

Figure S24. *Synchronous 2D correlation of transient kinetic step response switch at 175 °C on Co/CeO$_2$. Conditions: 0.6 mbar CO$_2$ / 200 mbar H$_2$/ balanced with He, total flow 200 ml/min, 175 °C.*

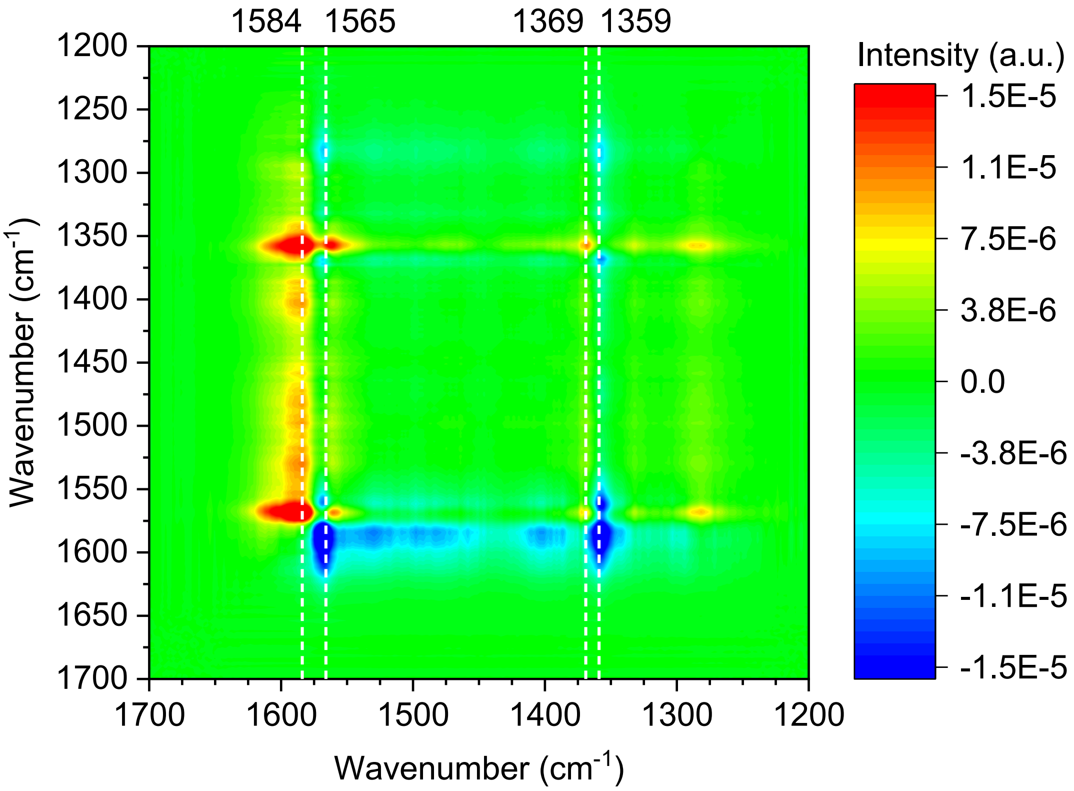

Figure S25. *Asynchronous 2D correlation of transient kinetic step response switch at 175 °C on Co/CeO$_2$. Conditions: 0.6 mbar CO$_2$ / 200 mbar H$_2$/ balanced with He, total flow 200 ml/min, 175 °C.*

Transient Operando FTIR Spectroscopy

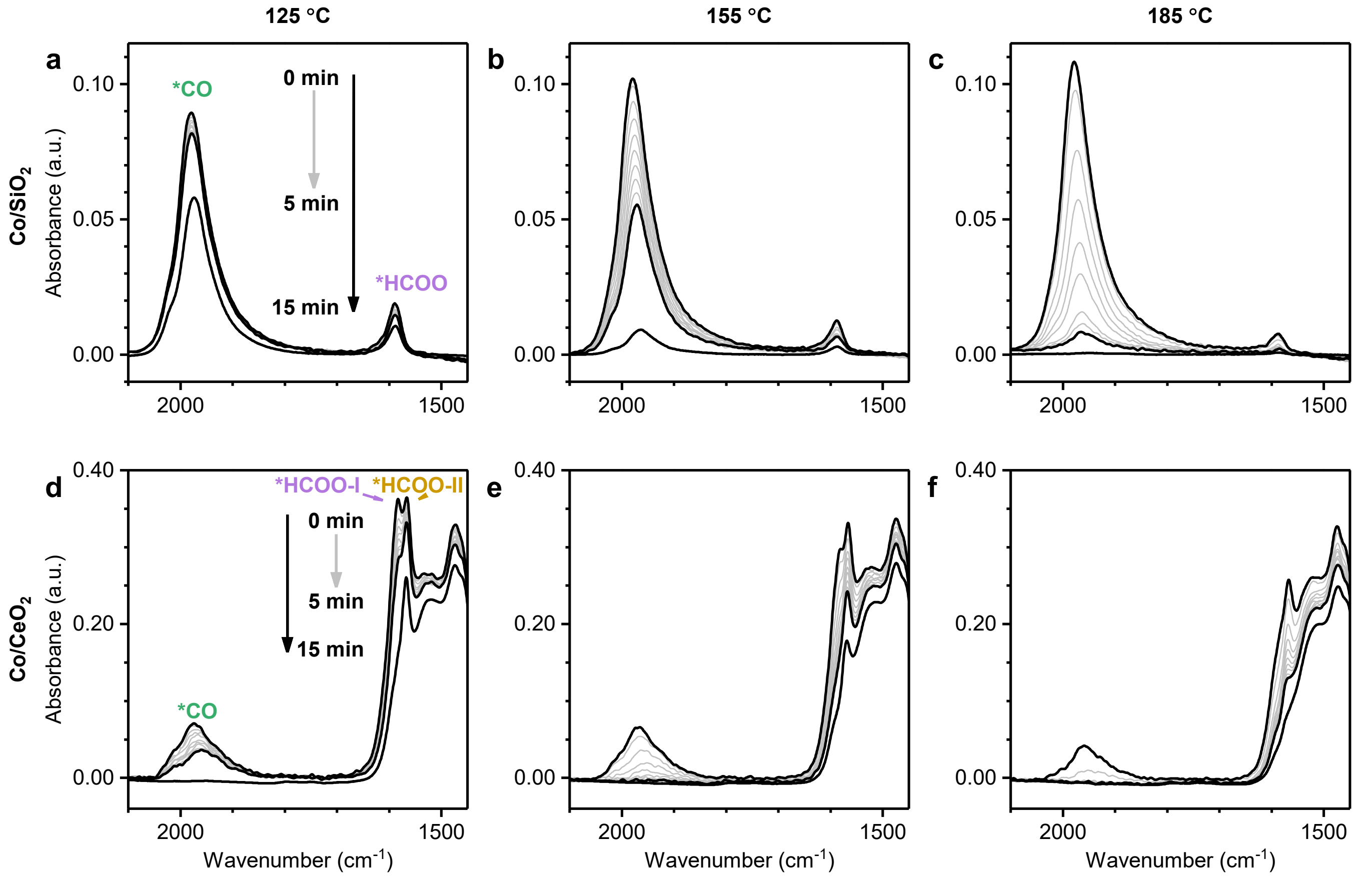

Figure S26. *Operando transient kinetic step response Fourier-transform infrared spectroscopy on a-c) Co/SiO₂ and d-f) Co/CeO₂. The CO_2/H_2/He to H_2/He switches at 125 °C (a, d), 155 °C (b, e) and 185 °C (c, f) demonstrate the reaction temperature dependence for the response of the surface species. Black bold lines indicate scans acquired at 0, 5, and 15 minutes. Gray lines show the decay in species in the first 5 minutes. Carbonyls are labeled by *CO and formates by *HCOO. Conditions: 0.6 mbar CO_2 / 200 mbar H_2 / balanced with He, total flow 200 ml/min, 125/155/185 °C.*

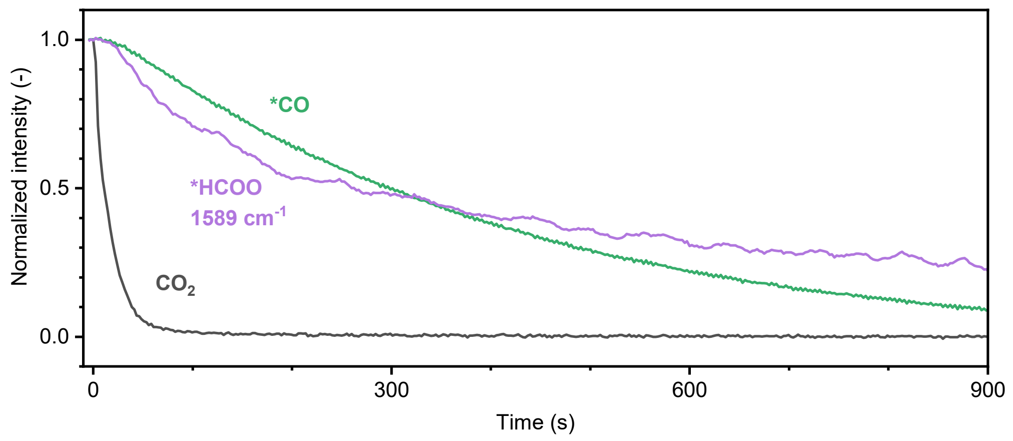

Figure S27. *Normalized step response Fourier-transform infrared spectroscopy on Co/SiO₂ for carbonyls and formates at 155 °C. The formate species on Co/SiO₂ display a fast initial decomposition rate followed by a period of slower decomposition. The decay of gaseous CO_2 is given as a comparison in black. Conditions: 0.6 mbar CO_2 / 200 mbar H_2 / balanced with He, total flow 200 ml/min, 125-185 °C.*

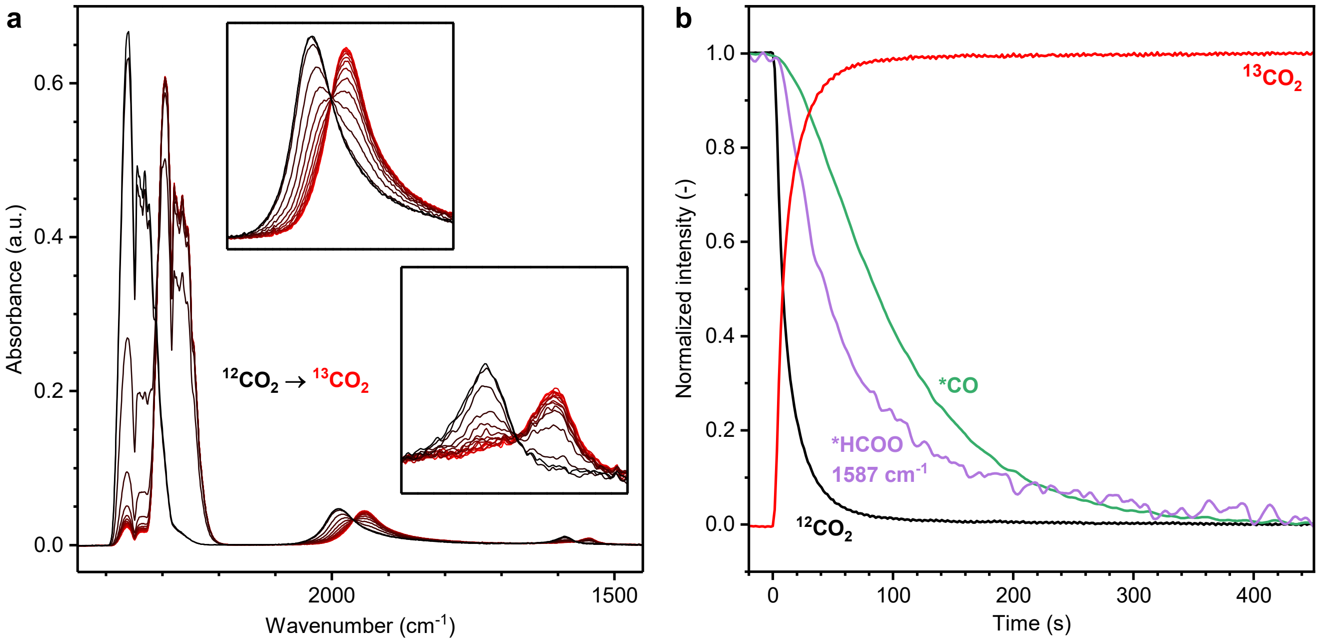

Figure S28. *Steady-state isotopic transient kinetic analysis on Co/SiO$_2$. a) Switch from $^{12}CO_2$ to $^{13}CO_2$. The inserts provide a zoom-in on the carbonyl and formate region. b) Normalized switch of Figure S28a for carbonyls and formates. The normalized responses demonstrate that the isotope exchange of the formates precedes that of the carbonyls, in line with the transient kinetic step response experiments. The decay of gaseous CO_2 is given as a comparison in black. Conditions: 25 mbar CO_2 / 200 mbar H_2/ balanced with He, total flow 200 ml/min, 175 °C.*

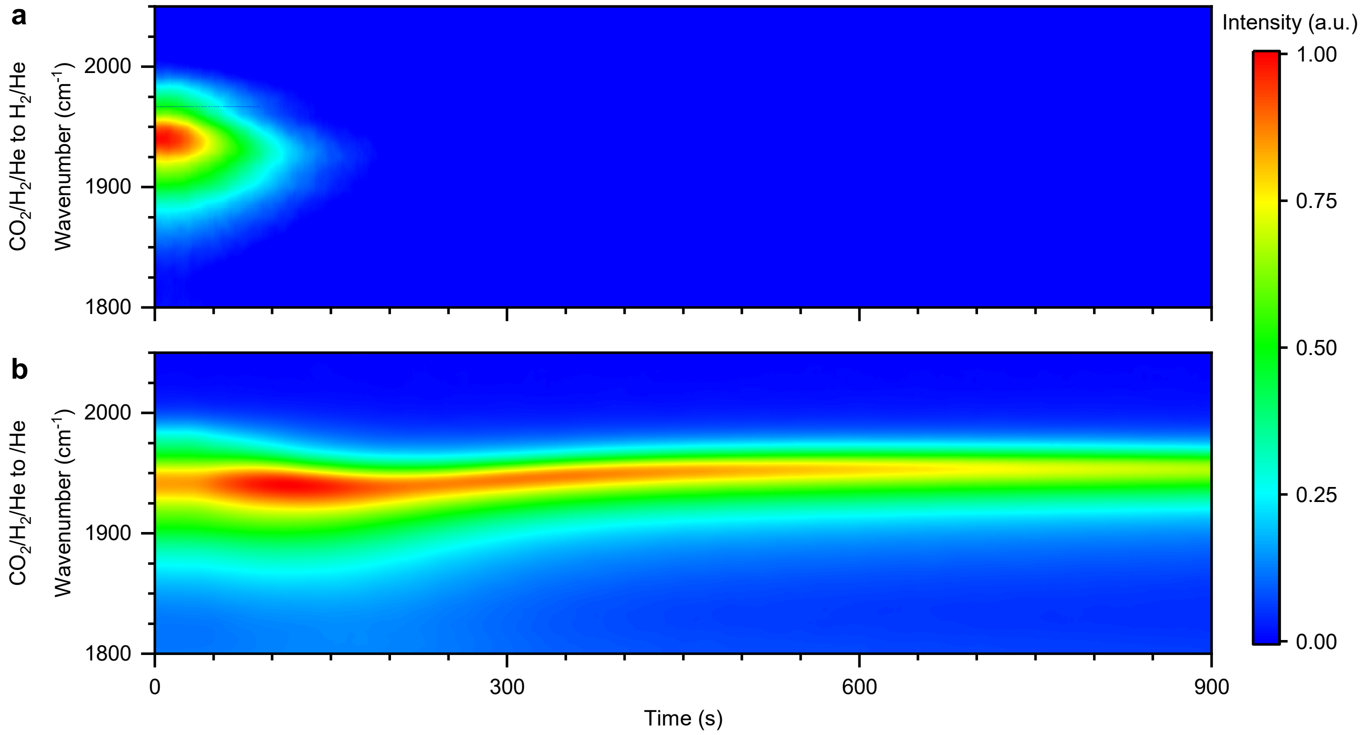

Figure S29. *Operando transient kinetic step response Fourier-transform infrared spectroscopy on Co/CeO$_2$. The feed was switched from $CO_2/H_2/He$ to H_2/He (a) and from $CO_2/H_2/He$ to He (b). When the feed is switched to H_2/He, the hydrogenation of *CO to CH_4 continues, resulting in a fast decay of the *CO bands intensity. In contrast, the CO decay is much slower in the absence of H_2 demonstrating that the decay observed in the operando transient kinetic step response experiments (switching to H_2/He) cannot be attributed to desorption of CO. Conditions: 0.6 mbar CO_2 / 200 mbar H_2/ balanced with He, total flow 200 ml/min, 155 °C.*

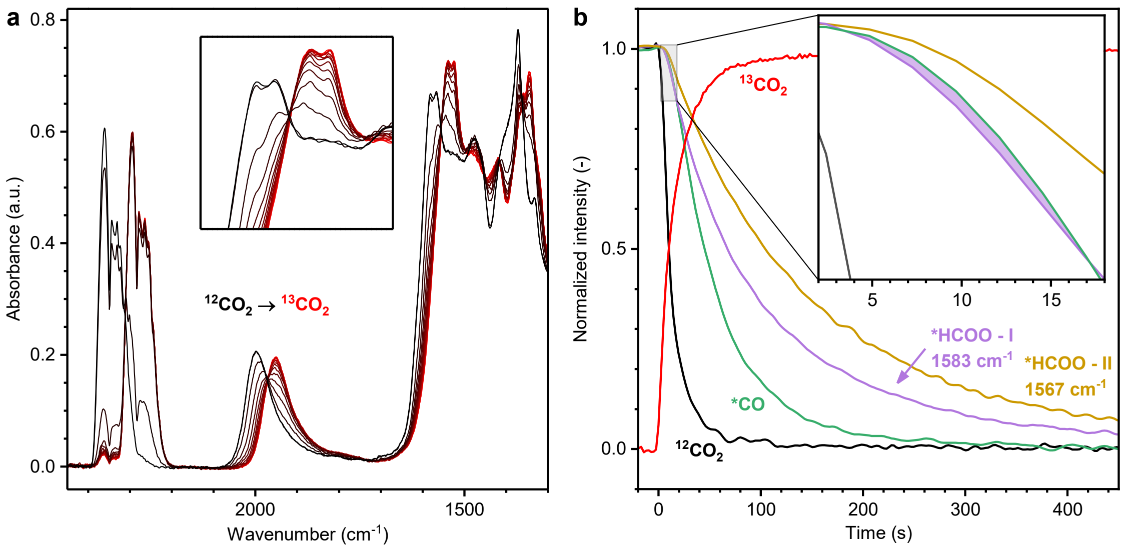

Figure S30. *Steady-state isotopic transient kinetic analysis on Co/CeO₂. a) Switch from* $^{12}CO_2$ *to* $^{13}CO_2$*. The inset provides a zoom-in on the formate region. b) Normalized switch of Figure S30a for carbonyls and both formates demonstrating that exchange of formate-I precedes the exchange of formate-II, in line with the transient kinetic step response experiments. Right after the switch the active formate (formate-I) precedes the carbonyl exchange as on Co/SiO₂ (inset). The decay of gaseous CO₂ is given as a comparison in black. Conditions: 25 mbar CO₂ / 200 mbar H₂/ balanced with He, total flow 200 ml/min, 175 °C.*

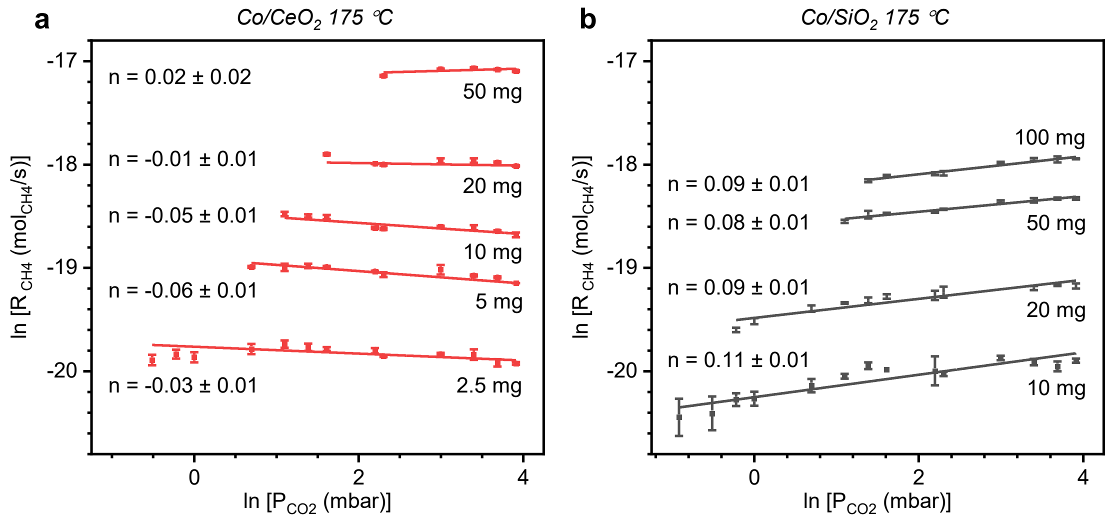

Figure S31. *Reaction orders with respect to* CH_4 *for a) Co/CeO₂ and b) Co/SiO₂ at 175 °C with CO₂ partial pressures varying from 0.4 to 5 mbar. The* CH_4 *reaction orders were significantly lower for the same catalyst loadings of Co/CeO₂ compared to Co/SiO₂, indicative of an effective adsorption and activation of CO₂ on Co/CeO₂. The error bars indicate the standard deviation between GC measurements (CO₂ conversions below 11%). For all measurements, He was used to obtain a total flow of 50 ml/min containing 20%* H_2 *besides CO₂. Catalyst loadings of 2.5, 5, 10, 20, and 50 mg for Co/CeO₂ and 10, 20, 50, and 100 mg for Co/SiO₂ were used.*

Note S5. Transient operando FTIR at different CO_2 partial pressures

The transient kinetic step response switches were compared at low and high partial pressures of CO_2 (Figure S32). Comparing the two catalysts at 25 mbar, it was again found that the carbonyl conversion was much faster on Co/CeO_2 (Figure S32a) compared to Co/SiO_2 (Figure S32b). Comparing the response of carbonyls and formates on Co/SiO_2 at both partial pressures (Figure S32b), the rates were very similar. On the contrary, the active species on Co/CeO_2 displayed a much longer response time at high partial pressures compared to low partial pressures (Figure S32a). Switching the feed from CO_2 / H_2 / He to H_2 / He at 25 mbar did not result in a steep, almost instant decrease for both the formates and carbonyls as for 0.6 mbar CO_2, but initially displayed a plateau at the higher partial pressure. It can be hypothesized that this plateau is caused by the large reservoir of carbon species formed from adsorbed CO_2 (e.g., carbonates and bicarbonates) on Co/CeO_2. This large carbon species reservoir, including the formates, can still supply activated CO_2 to the methane production pathway after the switch. The sustained methane production is supported by mass-spectrometry (Figure S6). Indeed, the absence of such a carbon reservoir on SiO_2 is in line with the very similar behavior observed at both partial pressures for SiO_2. Even at the higher partial of CO_2 of 25 mbar, methane production ceases right after the switch as judged from mass-spectrometry (Figure S33).

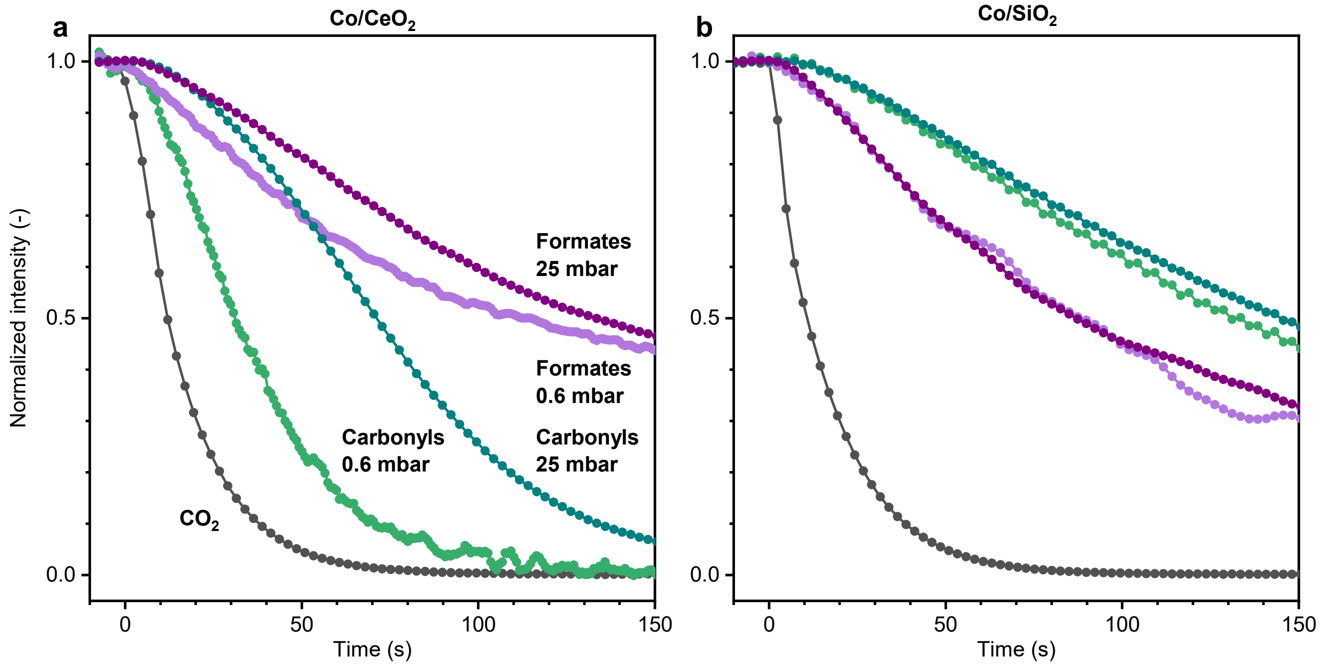

Figure S32. *Comparison of transient kinetic step response switches at 0.6 mbar and 25 mbar CO_2 on a) Co/CeO_2 and b) Co/SiO_2 at 175 °C. Conditions: 0.6 or 25 mbar CO_2 / 200 mbar H_2/ balanced with He, total flow 200 ml/min, 175 °C.*

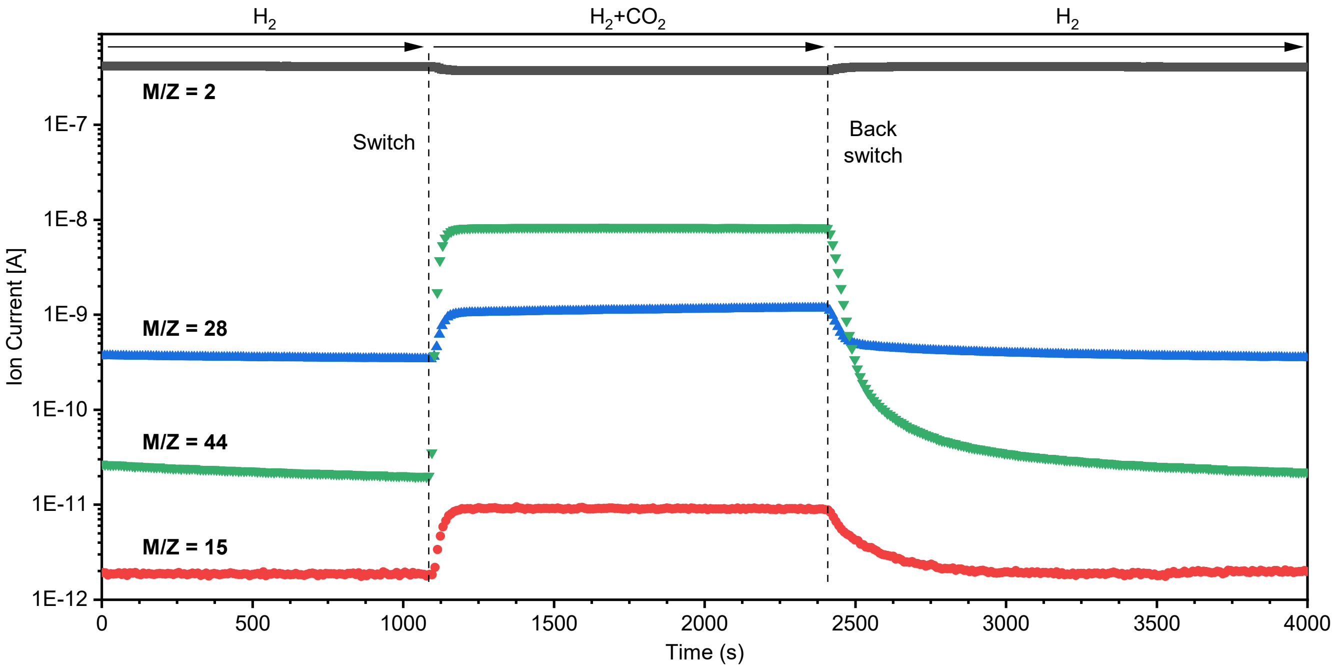

Figure S33. *Mass spectrometry data of the FTIR experiment at 25 mbar CO_2 for Co/SiO_2. Similar catalytic activity was observed before and after the switch from H_2 to CO_2+H_2. After the back switch, both the CO and CH_4 production (followed by m/z = 28 and 15, respectively) decreased concurrently with the CO_2 (m/z = 44). Conditions: 25 mbar CO_2 / 200 mbar H_2 balanced with He, total flow 200 ml/min.*

References

[1] L. Jiao, J. R. Regalbuto, *J. Catal.* **2008**, *260*, 329–341.
[2] I. Noda, Y. Ozaki, *Two-Dimensional Correlation Spectroscopy: Applications in Vibrational and Optical Spectroscopy*, Wiley, Chichester, West Sussex, England ; Hoboken, NJ, **2002**.
[3] I. Noda, *Appl. Spectrosc.* **1990**, *44*, 550–561.
[4] L. Artiglia, F. Orlando, K. Roy, R. Kopelent, O. Safonova, M. Nachtegaal, T. Huthwelker, J. A. van Bokhoven, *J. Phys. Chem. Lett.* **2017**, *8*, 102–108.
[5] T. Skála, F. Šutara, K. C. Prince, V. Matolín, *J. Electron Spectrosc. Relat. Phenom.* **2009**, *169*, 20–25.
[6] V. Muravev, G. Spezzati, Y.-Q. Su, A. Parastaev, F.-K. Chiang, A. Longo, C. Escudero, N. Kosinov, E. J. M. Hensen, *Nat. Catal.* **2021**, *4*, 469–478.
[7] M. C. Biesinger, B. P. Payne, A. P. Grosvenor, L. W. M. Lau, A. R. Gerson, R. St. C. Smart, *Appl. Surf. Sci.* **2011**, *257*, 2717–2730.
[8] S. Kato, M. Ammann, T. Huthwelker, C. Paun, M. Lampimäki, M.-T. Lee, M. Rothensteiner, J. A. van Bokhoven, *Phys. Chem. Chem. Phys.* **2015**, *17*, 5078–5083.
[9] A. Parastaev, V. Muravev, E. Huertas Osta, A. J. F. van Hoof, T. F. Kimpel, N. Kosinov, E. J. M. Hensen, *Nat. Catal.* **2020**, *3*, 526–533.
[10] G. Melaet, W. T. Ralston, C.-S. Li, S. Alayoglu, K. An, N. Musselwhite, B. Kalkan, G. A. Somorjai, *J. Am. Chem. Soc.* **2014**, *136*, 2260–2263.
[11] V. Iablokov, S. K. Beaumont, S. Alayoglu, V. V. Pushkarev, C. Specht, J. Gao, A. P. Alivisatos, N. Kruse, G. A. Somorjai, *Nano Lett.* **2012**, *12*, 3091–3096.
[12] S. K. Beaumont, S. Alayoglu, C. Specht, W. D. Michalak, V. V. Pushkarev, J. Guo, N. Kruse, G. A. Somorjai, *J. Am. Chem. Soc.* **2014**, *136*, 9898–9901.
[13] W. Li, X. Nie, X. Jiang, A. Zhang, F. Ding, M. Liu, Z. Liu, X. Guo, C. Song, *Appl. Catal. B Environ.* **2018**, *220*, 397–408.
[14] J. Díez-Ramírez, P. Sánchez, V. Kyriakou, S. Zafeiratos, G. E. Marnellos, M. Konsolakis, F. Dorado, *J. CO2 Util.* **2017**, *21*, 562–571.
[15] H. H. Shin, L. Lu, Z. Yang, C. J. Kiely, S. McIntosh, *ACS Catal.* **2016**, *6*, 2811–2818.
[16] G. Zhou, H. Liu, Y. Xing, S. Xu, H. Xie, K. Xiong, *J. CO2 Util.* **2018**, *26*, 221–229.
[17] T. Franken, J. Terreni, A. Borgschulte, A. Heel, *J. Catal.* **2020**, *382*, 385–394.
[18] C. Vogt, E. Groeneveld, G. Kamsma, M. Nachtegaal, L. Lu, C. J. Kiely, P. H. Berben, F. Meirer, B. M. Weckhuysen, *Nat. Catal.* **2018**, *1*, 127–134.
[19] D. Beierlein, D. Häussermann, M. Pfeifer, T. Schwarz, K. Stöwe, Y. Traa, E. Klemm, *Appl. Catal. B Environ.* **2019**, *247*, 200–219.
[20] P. A. U. Aldana, F. Ocampo, K. Kobl, B. Louis, F. Thibault-Starzyk, M. Daturi, P. Bazin, S. Thomas, A. C. Roger, *Catal. Today* **2013**, *215*, 201–207.
[21] C. K. Vance, C. H. Bartholomew, *Appl. Catal.* **1983**, *7*, 169–177.
[22] N. Perkas, G. Amirian, Z. Zhong, J. Teo, Y. Gofer, A. Gedanken, *Catal. Lett.* **2009**, *130*, 455–462.
[23] M. Yamasaki, H. Habazaki, K. Asami, K. Izumiya, K. Hashimoto, *Catal. Commun.* **2006**, *7*, 24–28.
[24] J.-N. Park, E. W. McFarland, *J. Catal.* **2009**, *266*, 92–97.
[25] S. Rahmani, M. Rezaei, F. Meshkani, *J. Ind. Eng. Chem.* **2014**, *20*, 1346–1352.
[26] W. L. Vrijburg, E. Moioli, W. Chen, M. Zhang, B. J. P. Terlingen, B. Zijlstra, I. A. W. Filot, A. Züttel, E. A. Pidko, E. J. M. Hensen, *ACS Catal.* **2019**, *9*, 7823–7839.
[27] W. L. Vrijburg, J. W. A. van Helden, A. Parastaev, E. Groeneveld, E. A. Pidko, E. J. M. Hensen, *Catal. Sci. Technol.* **2019**, *9*, 5001–5010.
[28] J. Li, Y. Lin, X. Pan, D. Miao, D. Ding, Y. Cui, J. Dong, X. Bao, *ACS Catal.* **2019**, *9*, 6342–6348.
[29] G. D. Weatherbee, C. H. Bartholomew, *J. Catal.* **1982**, *77*, 460–472.
[30] G. D. Weatherbee, C. H. Bartholomew, *J. Catal.* **1981**, *68*, 67–76.
[31] T. Van Herwijnen, H. Van Doesburg, W. A. De Jong, *J. Catal.* **1973**, *28*, 391–402.

[32] T. A. Le, T. W. Kim, S. H. Lee, E. D. Park, *Catal. Today* **2018**, *303*, 159–167.
[33] J. Liu, W. Bing, X. Xue, F. Wang, B. Wang, S. He, Y. Zhang, M. Wei, *Catal. Sci. Technol.* **2016**, *6*, 3976–3983.
[34] R. C. Reuel, C. H. Bartholomew, *J. Catal.* **1984**, *85*, 63–77.
[35] G. N. Vayssilov, M. Mihaylov, P. St. Petkov, K. I. Hadjiivanov, K. M. Neyman, *J. Phys. Chem. C* **2011**, *115*, 23435–23454.
[36] A. Paredes-Nunez, D. Lorito, N. Guilhaume, C. Mirodatos, Y. Schuurman, F. C. Meunier, *Catal. Today* **2015**, *242*, 178–183.
[37] A. Yu. Khodakov, J. Lynch, D. Bazin, B. Rebours, N. Zanier, B. Moisson, P. Chaumette, *J. Catal.* **1997**, *168*, 16–25.
[38] C. J. Weststrate, J. van de Loosdrecht, J. W. Niemantsverdriet, *J. Catal.* **2016**, *342*, 1–16.
[39] W. Chen, B. Zijlstra, I. A. W. Filot, R. Pestman, E. J. M. Hensen, *ChemCatChem* **2018**, *10*, 136–140.
[40] D. Song, J. Li, Q. Cai, *J. Phys. Chem. C* **2007**, *111*, 18970–18979.
[41] G. Kadinov, C. Bonev, S. Todorova, A. Palazov, *J. Chem. Soc. Faraday Trans.* **1998**, *94*, 3027–3031.
[42] F. M. T. Mendes, C. A. C. Perez, F. B. Noronha, C. D. D. Souza, D. V. Cesar, H. J. Freund, M. Schmal, *J. Phys. Chem. B* **2006**, *110*, 9155–9163.
[43] S. M. de Lima, A. M. da Silva, L. O. O. da Costa, U. M. Graham, G. Jacobs, B. H. Davis, L. V. Mattos, F. B. Noronha, *J. Catal.* **2009**, *268*, 268–281.
[44] P. A. Kumar, M. D. Tanwar, N. Russo, R. Pirone, D. Fino, *Catal. Today* **2012**, *184*, 279–287.
[45] C. Li, Y. Song, Y. Chen, Q. Xin, X. Han, W. Li, in *Stud. Surf. Sci. Catal.* (Eds.: C. Li, Q. Xin), Elsevier, **1997**, pp. 439–446.
[46] K. Zhao, M. Calizzi, E. Moioli, M. Li, A. Borsay, L. Lombardo, R. Mutschler, W. Luo, A. Züttel, *J. Energy Chem.* **2021**, *53*, 241–250.
[47] H.-K. Lin, C.-B. Wang, H.-C. Chiu, S.-H. Chien, *Catal. Lett.* **2003**, *86*, 63–68.
[48] S. A. Singh, G. Madras, *Appl. Catal. Gen.* **2015**, *504*, 463–475.
[49] L. B. Backman, A. Rautiainen, A. O. I. Krause, M. Lindblad, *Catal. Today* **1998**, *43*, 11–19.
[50] T. Sakpal, L. Lefferts, *J. Catal.* **2018**, *367*, 171–180.
[51] F. Zhang, Z. Liu, S. Zhang, N. Akter, R. M. Palomino, D. Vovchok, I. Orozco, D. Salazar, J. A. Rodriguez, J. Llorca, J. Lee, D. Kim, W. Xu, A. I. Frenkel, Y. Li, T. Kim, S. D. Senanayake, *ACS Catal.* **2018**, *8*, 3550–3560.
[52] K. Chang, H. Zhang, M. Cheng, Q. Lu, *ACS Catal.* **2019**, *10*, 613–631.
[53] T. Matsukawa, A. Hoshikawa, E. Niwa, M. Yashima, T. Ishigaki, *CrystEngComm* **2018**, *20*, 155–158.
[54] A. H. Clark, K. A. Beyer, S. Hayama, T. I. Hyde, G. Sankar, *Chem. Mater.* **2019**, *31*, 7744–7751.
[55] G. Vilé, B. Bridier, J. Wichert, J. Pérez-Ramírez, *Angew. Chem. Int. Ed.* **2012**, *51*, 8620–8623.
[56] C. Riley, S. Zhou, D. Kunwar, A. De La Riva, E. Peterson, R. Payne, L. Gao, S. Lin, H. Guo, A. Datye, *J. Am. Chem. Soc.* **2018**, *140*, 12964–12973.
[57] J. Moon, Y. Cheng, L. L. Daemen, M. Li, F. Polo-Garzon, A. J. Ramirez-Cuesta, Z. Wu, *ACS Catal.* **2020**, *10*, 5278–5287.
[58] Z. Li, K. Werner, K. Qian, R. You, A. Płucienik, A. Jia, L. Wu, L. Zhang, H. Pan, H. Kuhlenbeck, S. Shaikhutdinov, W. Huang, H.-J. Freund, *Angew. Chem. Int. Ed.* **2019**, *58*, 14686–14693.
[59] Z. Wu, Y. Cheng, F. Tao, L. Daemen, G. S. Foo, L. Nguyen, X. Zhang, A. Beste, A. J. Ramirez-Cuesta, *J. Am. Chem. Soc.* **2017**, *139*, 9721–9727.
[60] K. Sohlberg, S. T. Pantelides, S. J. Pennycook, *J. Am. Chem. Soc.* **2001**, *123*, 6609–6611.
[61] D. Schweke, L. Shelly, R. Ben David, A. Danon, N. Kostirya, S. Hayun, *J. Phys. Chem. C* **2020**, *124*, 6180–6187.
[62] C. Copéret, D. P. Estes, K. Larmier, K. Searles, *Chem. Rev.* **2016**, *116*, 8463–8505.
